# Structure Selection in Ionic Colloidal Crystals via Independent Charge Tuning

**DOI:** 10.1021/jacs.6c08143

**Published:** 2026-10-05

**Authors:** Steven van Kesteren, Shihao Zang, Cheuk W. Leung, Glen M. Hocky, Stefano Sacanna

**Affiliations:** † Department of Chemistry, 5894 New York University, New York, New York 10003, United States; ‡ Department of Information Technology and Electrical Engineering, ETH Zurich, Zurich 8092, Switzerland; § Simons Center for Computational Physical Chemistry, New York University, New York, New York 10003, United States

## Abstract

Colloidal building blocks can assemble into many different crystal structures, yet selecting a specific lattice without redesigning the particles remains a central challenge in programmable self-assembly. Here we demonstrate that independent tuning of colloidal surface charge enables deterministic structure selection using a fixed pair of oppositely charged colloids. Micromolar additions of simple ionic surfactants independently adjust the surface charge of each colloidal species without significantly altering solution ionic strength, allowing the same building blocks to assemble into Th_3_P_4_-, CsCl-, Cu_3_Au-, and Li_3_Fe-like crystals, the last of which represents a previously unreported colloidal crystal structure. Experiments and molecular dynamics simulations reveal that structure selection is governed by the competition between Coulombic attraction and lattice-specific like-charge repulsion. Experiments across different particle-size ratios further demonstrate the generality of this approach, with additional surfactant-induced transitions between Th_3_P_4_-, AlB_2_-, and Cr_3_Si-like structures. These findings establish charge asymmetry as a general design parameter for ionic colloidal assembly.

## Graphical abstract

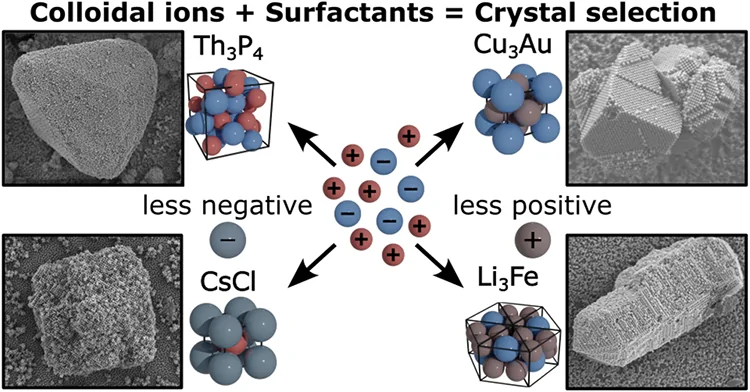

## Introduction

Structure selection in colloidal crystals is most commonly controlled through particle geometry, including size ratio, shape, and mixing ratio, which determine packing constraints and coordination environments accessible to a given particle pair.^1–4^ Because these geometric parameters are fixed once particles are synthesized, accessing different crystal structures typically requires designing and synthesizing new building blocks. An appealing alternative strategy would be to control structure selection by tuning interparticle interactions rather than particle geometry, but practical methods to do so remain limited.

In contrast to atomic ionic solids, where crystal structures are constrained by discrete valence states and strict charge neutrality, colloidal particles offer continuous tunability of both particle size and surface charge. This continuous tunability opens a much broader structural design space that includes crystal symmetries with no direct atomic counterpart.^5–8^ While geometric control of colloidal crystal structures has been extensively explored, the role of charge asymmetry between oppositely charged species as a control parameter for structure selection remains largely unexplored.

In oppositely charged colloidal systems, electrostatically-driven self-assembly is governed by a balance between Coulombic attraction and like-charge repulsion, both mediated by the electric double layer.^9^ Harnessing these forces to assemble ordered colloidal crystals requires a delicate energetic balance: attraction must be strong enough to stabilize the lattice, yet weak enough to allow particles to rearrange and anneal into their lowest-energy configurations.^4,10–12^ A central challenge in these systems is the tendency of oppositely charged particles to aggregate irreversibly when attraction dominates, preventing the formation of ordered structures. To address this, previous work has employed dielectrically matched solvents or grafted neutral polymer brushes to attenuate electrostatic and van der Waals attractions, enabling the formation of well-ordered ionic colloidal crystals.^1,2^

Electrostatic interactions can be modulated in two main ways: by changing ionic strength or by modifying particle surface charge. Adjusting salt concentration provides a straightforward screening mechanism, but it simultaneously affects both attraction and repulsion and therefore acts as a global control parameter.^9,13^ As a result, salt tuning alone provides limited control over structure selection because it does not allow independent tuning of the interactions experienced by each colloidal species. A more powerful strategy would be to independently tune the surface charge of each species, thereby directly controlling the balance between Coulombic attraction and structure-specific like-charge repulsion that ultimately determines crystal stability.

Here we demonstrate that simple ionic surfactants provide precisely this capability. By selectively adsorbing onto oppositely charged colloidal particles, sodium dodecyl sulfate (SDS) and cetyltrimethylammonium bromide (CTAB) allow independent and continuous tuning of the effective surface charge of each colloidal species. When combined with polymer-attenuated Coulombic self-assembly (PACS) interactions,^2^ this selective charge tuning enables deterministic selection among multiple crystal structures using a single pair of building blocks. Specifically, we access multiple crystal structures, including a previously unreported Li_3_Fe-like colloidal crystal, using a single pair of oppositely charged polystyrene colloids with independently tunable surface charge. Experiments and molecular dynamics simulations show that structure selection is governed by the competition between Coulombic attraction and lattice-specific like-charge repulsion, establishing charge asymmetry as a predictive design parameter for ionic colloidal assembly.

## Results and Discussion

To explore how electrostatic tuning dictates structural selection, we utilize the PACS framework.^2^ In this system, the strong attraction between oppositely charged colloids is moderated by a neutral polymer brush (here Pluronic F108) and a tunable Debye screening length.

Previous studies have shown that a wide range of crystal structures can be accessed by varying particle size ratio and composition.^2,7,14^ However, these parameters are fixed once the building blocks are synthesized, making it difficult to explore multiple structures within a single system.

Here, instead of modifying geometric or stoichiometric parameters, we achieve crystal structure selection by tuning the effective surface charges of the colloidal ions through the selective adsorption of ionic surfactants. Specifically, the cationic surfactant cetyltrimethylammonium bromide (CTAB) reduces the magnitude of the negative colloidal charge, while the anionic surfactant sodium dodecyl sulfate (SDS) reduces the magnitude of the positive colloidal charge.^15^

For all experiments, we use spherical polystyrene colloids with fixed geometry, corresponding to a positive-to-negative size ratio of 0.85 (204 nm (+) and 240 nm (−) diameters) and a number ratio of 3.2:1. Under these conditions, selective charge-specific adsorption serves as the primary control parameter for structure selection.

To ensure experimental reproducibility, all colloidal suspensions were pre-equilibrated with surfactant, salt, and Pluronic F108 prior to mixing. Crystallization was monitored in glass capillaries using two complementary protocols. Sealed capillaries were first used to probe the interaction energy landscape under fixed ionic strength conditions. To determine equilibrium crystal structures, we then employed a dialysis protocol in which the salt concentration is gradually reduced over time. This approach drives crystallization near the free-energy boundary for nucleation and allows particles to anneal into their lowest-energy configurations.^7^

### Selective Charge Tuning

To determine how CTAB and SDS modify the interaction landscape, we quantified their effect on the surface charge of each colloidal species. As shown in Figure 1, these surfactants do not act as simple screening agents; instead, they selectively adsorb onto oppositely charged particles, introducing a controlled charge asymmetry between the two species.

**Figure 1:**
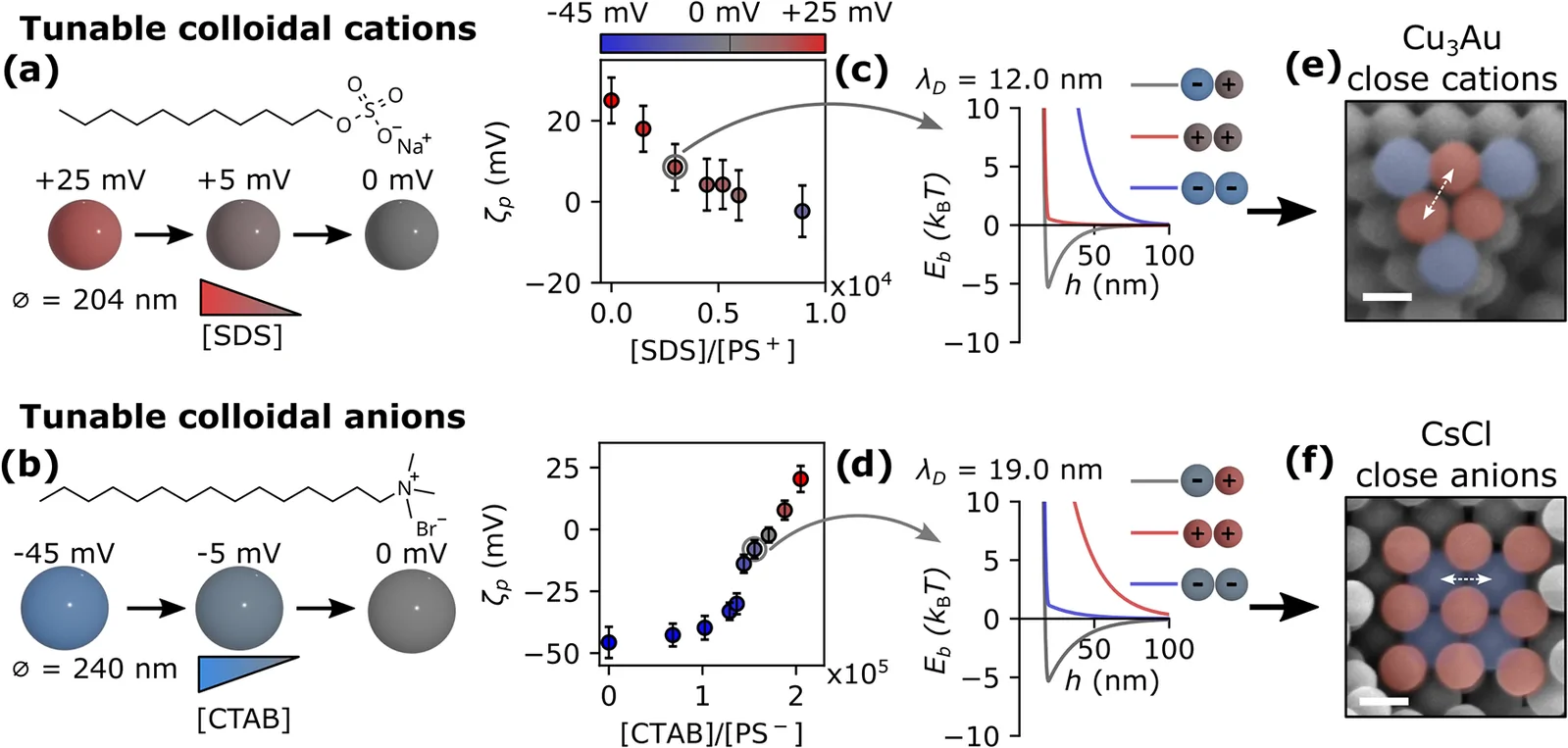
Selective charge tuning controls interaction asymmetry and lattice selection in ionic colloidal crystals. (a, b) The ζ-potential measurements showing selective charge attenuation of colloidal ions by oppositely charged surfactants. The addition of CTAB reduces the magnitude of the negative surface charge, while SDS reduces the magnitude of the positive surface charge. (c, d) DLVO-based interaction calculations illustrating how selective charge attenuation modifies the balance between attraction and like-charge repulsion. For the same attractive interaction strength, charge attenuation of one species shifts the system into regimes where either anion–anion or cation–cation repulsion dominates. (e, f) Because different crystal structures place like-charged particles at different separations, interaction asymmetry favors different lattice types. Structures such as CsCl minimize cation–cation contacts but place anions in close proximity, whereas structures such as Cu_3_Au favor close packing of cations. Selective charge tuning, therefore, enables structure selection by controlling which like-charge repulsion dominates. The scale bars are 200 nm.

ζ-potential measurements (Figure 1a,b) show that SDS monotonically reduces the magnitude of the positive surface charge, while CTAB selectively reduces the magnitude of the negative surface charge, with both species approaching apparent charge neutrality at sufficiently high surfactant concentrations. Qualitatively similar trends are observed for Pluronic F108-coated particles, although the absolute ζ-potential values are reduced by the presence of the polymer brush.^16^ The small shift in the concentration where the particle is apparently neutral likely arises from differences in ion accessibility within the polymer brush (Supporting Figure S1). Importantly, within the concentration range relevant to crystallization, no measurable change in surface charge is detected when CTAB is added to positively charged colloids or SDS to negatively charged ones, confirming that adsorption is selective for oppositely charged surfaces. Because ζ-potential measurements were performed at particle concentrations roughly two orders of magnitude below those used in crystallization experiments, these measurements are used to verify the direction and relative magnitude of charge modulation rather than to determine absolute surface charge values.

Reducing the surface charge of one colloidal species weakens the effective Coulombic attraction between oppositely charged particles. Within the PACS interaction framework, this reduction can be compensated by increasing the Debye length–that is, by lowering the salt concentration–to restore the interaction strength required for assembly (Figure 1c,d).

Consistent with this prediction, Figure 2a,b shows that reducing the magnitude of either surface charge leads to a systematic shift in the Debye length at which crystallization occurs in both experiments and simulations. With PACS interactions, crystallization occurs only within a narrow window of Debye lengths that is bounded at short range by a region where particles maintain a stable gas-like dispersion, and at longer range by a region exhibiting disordered aggregation (Figure 2c). We emphasize that only micromolar additions of surfactant are needed to shift the crystallization window to larger Debye lengths, corresponding to a reduction of up to 2 mM in the NaCl concentration required for crystallization. Because the surfactant concentrations are much lower than the salt concentration, their contribution to ionic strength is negligible, and Debye lengths are estimated from the NaCl concentration alone. At sufficiently high surfactant concentrations, suspensions remain stable even at long Debye lengths, consistent with the particles approaching charge neutrality (Figure 2a–c).

**Figure 2:**
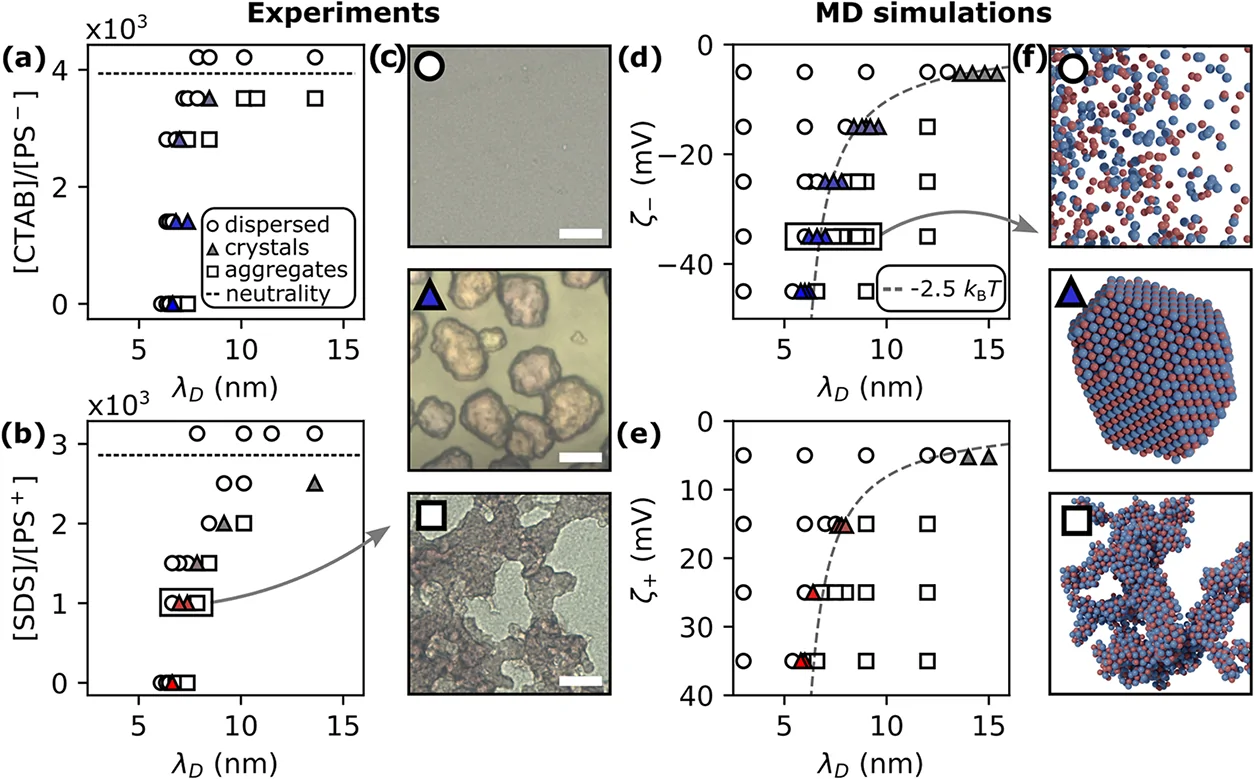
Critical crystallization salt concentrations can be controlled through addition of CTAB or SDS. (a, b) Experimental phase diagrams showing colloidal gas (circles), gels (squares), and crystals (triangles) as a function of Debye length for varying concentrations of CTAB (a) and SDS (b). The dotted line indicates the boundary above which the particles carry like charges. (c) Representative micrographs of the three phases: gas (top), crystals (middle), and gels (bottom). (d, e) MD simulations described in the main text predict phase diagrams showing colloidal gas (circles), aggregates (squares), and crystals (triangles) as a function of Debye length for different surface charges. The dotted line corresponds to an average potential energy per particle of −2.5 *k*_B_ *T*. In (d), the surface potential of the negative particles is varied while the positive particles are fixed at +35 mV; in (e), the surface potential of the positive particles is varied while the negative particles are fixed at −45 mV. (f) Renderings of the simulated phases showing gas (top), crystals (middle), and aggregates (bottom).

To relate these observations to interaction energies, we performed MD simulations of charged colloids within the PACS framework using our recently developed PACSim platform.^17^ Independently reducing the positive or negative surface charge–corresponding to the addition of SDS or CTAB, respectively–reproduces the experimentally observed shift of the crystallization window toward larger Debye lengths (Figure 2d,e). Across all conditions examined, crystallization occurs when the total potential energy per particle reaches approximately −2.5 *k*_B_ *T*, providing both an estimate of the interaction strength required for assembly and an approximate mapping between experimental Debye lengths and surface charge values at crystallization-relevant particle concentrations. Notably, whereas salt concentration enters the interaction potential nonlinearly–through an exponential dependence on the square root of ionic strength–the interaction energy scales linearly with the surface charge of each particle. This linearity makes surfactant-mediated charge tuning a particularly effective and fine-grained control strategy. This strategy is not restricted to the present chemistry: we have previously used a photoswitchable ionic surfactant to tune the surface charge of both polystyrene and fluorinated-polymer colloids,^18^ indicating that charge asymmetry, rather than the particular molecule used to impose it, is the operative design variable.

### Crystal Lattice Selection

Selective tuning of the colloidal surface charges leads to deterministic switching among several distinct ionic colloidal crystal structures. In the absence of surfactants, the system predominantly forms Th_3_P_4_-like crystals (Figure 3, top row). Reducing the negative surface charge magnitude (tuning the larger colloidal anion) with CTAB consistently stabilizes the CsCl-like structure (Figure 3, second row), whereas reducing the positive surface charge magnitude (tuning the smaller colloidal cation) with SDS produces Cu_3_Au-like lattices (Figure 3, third row) and Li_3_Fe-like lattices (Figure 3, fourth row and examined in more detail below). These structures typically coexist, but the relative occurrence of these two structures depends on SDS concentration: Li_3_Fe-like crystals are more frequently observed at lower surfactant loadings (∼1000–2000 SDS molecules per colloid, i.e., [SDS]/[PS^+^]), while Cu_3_Au-like structures dominate at higher loadings (∼2000–4000 SDS molecules per colloid).

**Figure 3:**
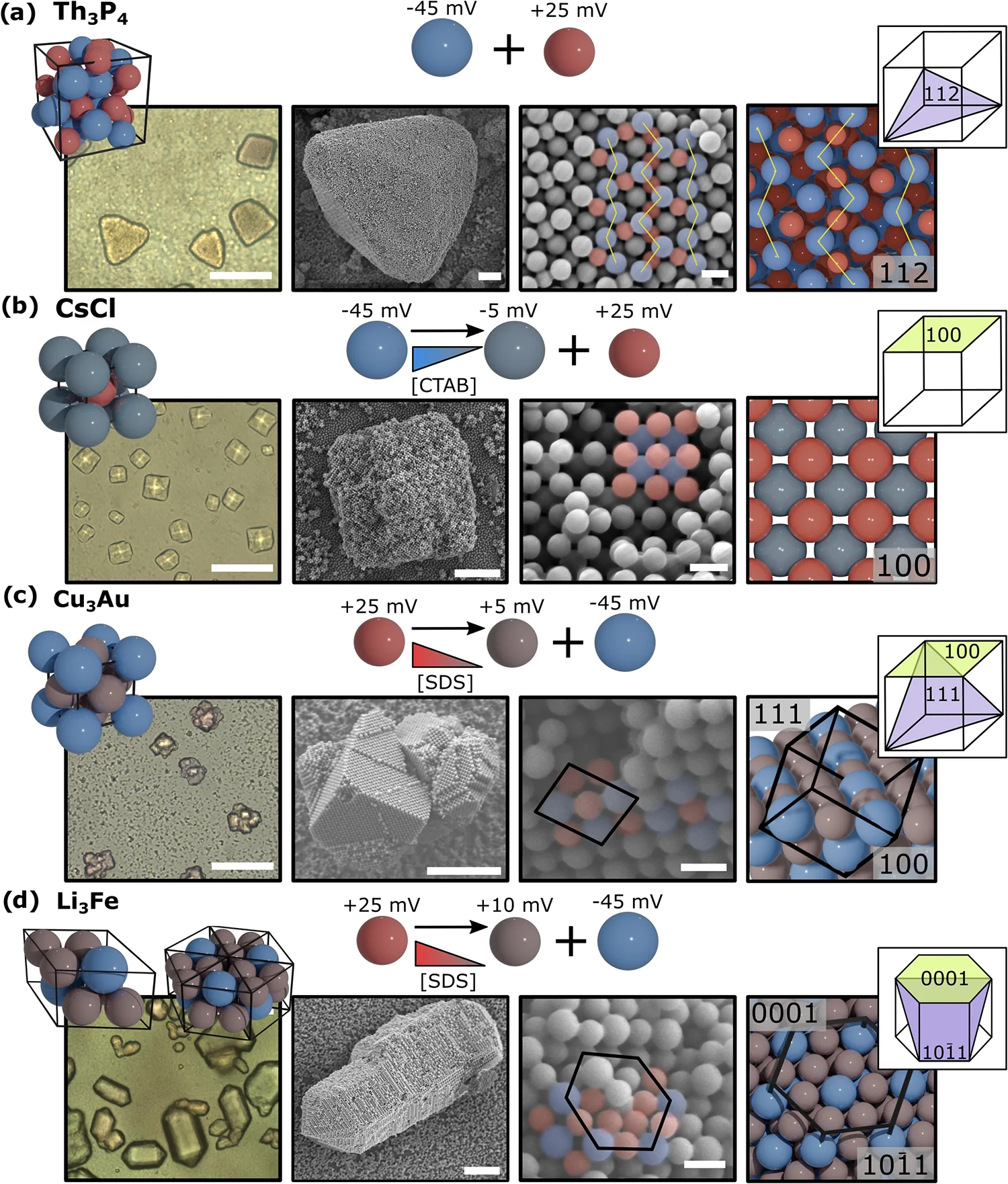
Crystal structure identification controlled by CTAB and SDS. Th_3_P_4_-, CsCl-, Cu_3_Au-, and Li_3_Fe-like crystals (a–d, respectively) are deterministically assembled from a single mixture of oppositely charged colloids by tuning their relative surface charges. For each structure (left to right): unit cell, brightfield micrograph in water, SEM micrographs of fixed crystals at increasing magnification with positively (red) and negatively (blue) charged colloids highlighted in false color, and 3D renderings of lattice planes corresponding to the SEM images with associated Miller indices. Scale bars: 50 μm (brightfield), 5 μm (low-magnification SEM), and 300 nm (high-magnification SEM).

To facilitate the assignment of these crystal structures, well-faceted crystals of sufficient size were required. While observing the overall morphology during crystallization at a fixed Debye length clearly distinguishes among colloidal gas, gel, and crystalline phases (Figure 2c), the resulting crystals were often too small or poorly developed for unambiguous lattice assignment. We therefore employed a dialysis protocol using a long capillary containing the oppositely charged colloidal suspension. In this protocol, we seal one end and leave the other open to a surfactant-matched, salt-free reservoir, resulting in a gradual and spatially controlled reduction in salt concentration along the capillary length. This approach slowly increases the effective electrostatic attraction, enabling controlled nucleation and the growth of large, well-defined colloidal crystals.

To preserve the assembled structures for imaging, the crystals were subsequently fixed by removing additional salt and surfactant, followed by a moderate thermal treatment. The progressive reduction in ionic strength increases the attractive interparticle potential, while subsequent heating partially softens the polystyrene particles, creating physical linkages that maintain structural integrity during drying and allowing SEM imaging (Figure 3). From SEM characterization, we identify Th_3_P_4_-, CsCl-, Cu_3_Au-, and Li_3_Fe-like crystals as described below.

Th_3_P_4_-, CsCl-, and Cu_3_Au-like crystals have all been reported previously in ionic or DNA-programmable colloidal systems,^1,7,14,19–22^ and are identified here on the basis of their characteristic crystal habits, facet geometries, and projected lattice planes.

Th_3_P_4_-like crystals exhibit a truncated triakis tetrahedron habit bounded by equivalent {112} facets, which display a distinct zig-zag arrangement of negatively charged colloids (Figure 3a). CsCl-like crystals are identified by their pyramidal morphologies, consistent with templated nucleation of positively charged particle layers at negatively charged glass surfaces. Minor hoppering exposes the (100) plane of the CsCl unit cell (Figure 3b). Cu_3_Au-like crystals appear as twinned cubic assemblies, a characteristic feature of this structure.^14,23^ The (100) surface plane is readily resolved at the single-particle level; although this alternating square motif can also arise in other cubic lattices, such as NaCl-like, simultaneous observation of the (100) and (111) planes, together with the 3:1 stoichiometry, uniquely identifies the structure as Cu_3_Au-like (Figure 3c, Supporting Figure S2).

Among the observed structures, the Li_3_Fe-like lattice represents, to our knowledge, a previously unreported colloidal crystal which we therefore characterize in detail in Figure 4. The assemblies form hexagonal prisms terminated by pyramidal caps, consistent with a hexagonal unit cell and reflected in the macroscopic crystal habit (Figure 4a). The basal (0001) plane forms the top facet, while six equivalent (101̅1) facets truncate the prism at the tips and the (101̅0) facets define the lateral faces of the prismatic body; occasional (112̅0) facets are also observed (Supporting Figure S3). The (101̅0) faces exhibit a corrugated surface composed of protruding rows and grooves that readily accommodate random free particles, which prevents clean, flat SEM imaging, whereas the pyramidal (101̅1) facets clearly reveal step edges between successive layers.

**Figure 4:**
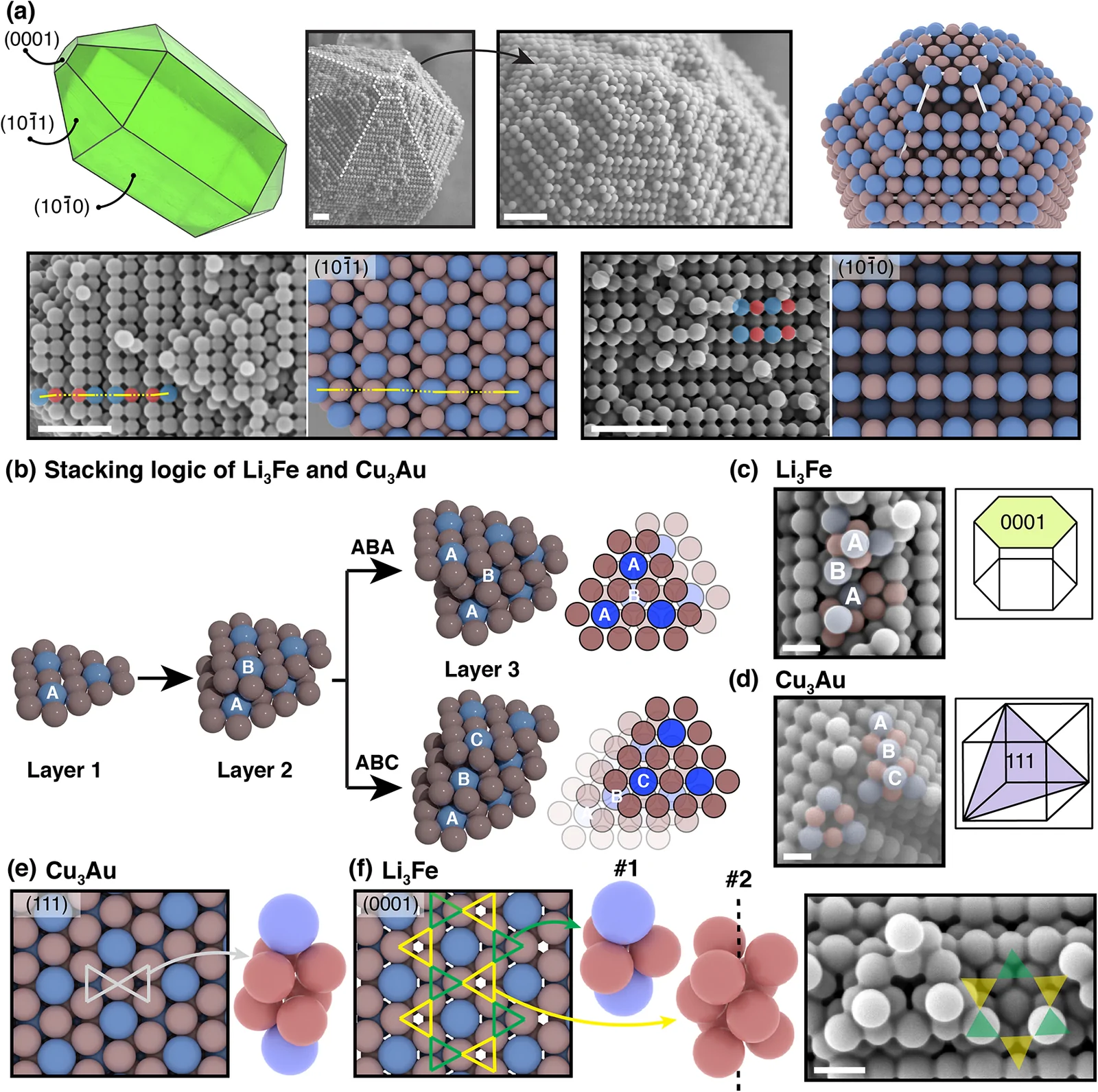
Characterization of the novel Li_3_Fe-like crystals. (a) The Li_3_Fe hexagonal prismatic habit exposes three characteristic planes: the basal (0001) plane, equivalent (101̅1) facets truncating the prism tips, and (101̅0) facets forming the lateral faces of the prism. The morphology and planes observed in SEM match the 3D renderings generated from the stacking of the Li_3_Fe unit cell (Supporting Figure S3). In the SEM micrographs, annotations and positive (red) and negative (blue) colloids in false colors highlight key surface features. On the (101̅1) facets, dashed yellow lines mark step edges. On the (101̅0) facets, protruding rows form extended stripe-like features, with the grooves between them readily accommodating random free particles. (b–f) The different stacking logic of Li_3_Fe- and Cu_3_Au-like crystals. (b) The hexagonal stacking orders of a shared common plane for Li_3_Fe­(0001) and Cu_3_Au­(111) are shown schematically. In Li_3_Fe, the third layer stacks at the same position as the first layer (ABA), while in Cu_3_Au, the third layer stacks at a position shifted relative to the first layer (ABC). (c, d) SEM micrographs with stacking planes labeled by letters. The (0001) plane is shown for Li_3_Fe-like crystals (c), and the (111) plane for Cu_3_Au-like crystals (d). (e, f) The stacking order of Li_3_Fe and Cu_3_Au results in different local environments. Cu_3_Au has only one repeating local environment in the (111) plane, as shown in grey in (e). Li_3_Fe has two local environments in (f): triangles of small particles between layers of large particles (green), and columns of such triangles extending along the *c* axis in alternating orientations (yellow), the latter defining partially open channels through the structure. The included SEM micrograph indicates that the spacing of the first environment (green) is smaller than the second environment (yellow). The scale bars are 1 μm for (a) and 300 nm for (c, d, f).

The basal (0001) plane exhibits an in-plane motif similar to the (111) plane of Cu_3_Au, consistent with a 3:1 stoichiometry, but the stacking sequence distinguishes the two structures. In Li_3_Fe, close-packed planes follow an ABAB stacking sequence characteristic of hexagonal symmetry (space group *P*6_3_/*mmc*), whereas Cu_3_Au exhibits ABCABC stacking associated with face-centered cubic order (Figure 4b–d). This difference in stacking arrangement makes otherwise similar planar motifs distinct: in Cu_3_Au, all nearest-neighbor distances between the small particles are identical (Figure 4e), whereas in Li_3_Fe two distinct local environments emerge. One consists of triangular arrangements confined between neighboring layers of large particles (green in Figure 4f), while the other forms columns of such triangles extending along the *c* axis in alternating orientations (yellow in Figure 4f). These columns define partially open channels through the structure of only small particles. This suggests that transport through the lattice may be directionally dependent, with the *c* axis providing a more continuous pathway. In addition, the presence of both confined and connected regions could allow for simple size- or interaction-based selectivity, for example, in transport or filtration.

We wondered whether this newly obtained structure might have interesting optical properties. To check this, we computed the photonic band structure, and while calculations do not show a complete band gap even at high dielectric contrast (ϵ = 16.0, Supporting Figure S4),^24^ we find that the structure may still support more limited or directional optical effects due to its increased anisotropy. Overall, the Li_3_Fe lattice can be viewed as a testament to the versatility of ionic colloidal crystals and may be relevant for applications requiring directional transport or selective permeability.

### Crystal Stability

The surfactant-controlled switching between Th_3_P_4_-, CsCl-, Cu_3_Au-, and Li_3_Fe-like crystals observed experimentally motivates a computational investigation of the energetic factors governing structure selection. While standard MD simulations of colloidal assembly capture experimentally observed CsCl- and Th_3_P_4_-like lattices, quantitative comparison between simulated and experimental assembly conditions reveals some discrepancies. For example, MD simulations performed at a positive-to-negative number ratio of 1:1 successfully nucleate CsCl- and Th_3_P_4_-like crystals, whereas experiments at this ratio show no assembly and instead require ratios above ∼1.5 unless CTAB is added (Supporting Figure S5). Furthermore, in simulations, a positive-to-negative number ratio of 3:1 results in no crystal nucleation within a reasonable computation time (48h on a high-end GPU).

We attribute these discrepancies to known experimental and computational constraints rather than to limitations of the interaction model. In experiments, preferential adsorption of positively charged particles onto glass substrates effectively reduces their concentration in suspension. In simulations, the accessible time scales are significantly shorter, and particle concentrations are elevated to promote nucleation, which can favor aggregation. Together, these differences account for the observed discrepancies in crystallization behavior.

More broadly, colloidal crystallization pathways depend sensitively on substrates^2^ and on the history of interactions during assembly.^7,18^ As a result, simulations of assembly alone are difficult to interpret without direct comparison to experiments. We therefore sought to leverage our theoretical PACS model to help us predict which structure would appear under which conditions, and developed a complementary stability analysis framework that tests the energetic stability of candidate lattices under specified interaction parameters (full details in Methods section).

In this approach, ideal crystals are initialized from their unit cells at lattice spacings that minimize the total interaction energy and then equilibrated at finite temperature. Rather than predicting assembly pathways, this method tests whether a given lattice remains stable once formed, effectively probing stability from the final state. Similar approaches have been implemented using Monte Carlo simulations^6^ or analytical models;^5^ here, however, we directly relate stability trends to experimentally tunable surface charges and Debye screening lengths. This framework is most informative when combined with experiments that establish which structures are accessible. Although it does not predict assembly pathways or absolute free energies, it allows us to assess whether specific crystal structures are stable under the PACS interaction potential and to map approximate (meta)­stability boundaries as a function of surface charge and screening length. We emphasize that this is an extremely fast (tens of seconds per structure on a laptop) and easy-to-perform calculation that can quickly inform experimental design. We have recently implemented full thermodynamic integration from a reference crystal^25,26^ in PACSim, and those calculations, while tractable, are orders of magnitude more expensive and have so far given qualitatively similar results, since for most cases the enthalpy dominates over entropic effects. Alternatively, it is possible to perform seeded growth by carving an initial seed from our equilibrated bulk structures to assess whether growth is favored under dilute conditions, but we reserve those more expensive calculations for future studies and focus here on our theory-guided experiments.

Using our fast stability analysis, we map structural stability across the parameter space of varying surface charge and Debye length, revealing trends that closely mirror the experimental observations (Figure 5). Th_3_P_4_ remains stable across the broadest range of surface charges, whereas CsCl is stabilized when the colloidal anion surface charge is reduced (corresponding to CTAB addition) and destabilized when the colloidal cation charge decreases. Conversely, Li_3_Fe and Cu_3_Au exhibit enhanced stability at reduced colloidal cation charge (corresponding to SDS addition) and become unstable when the colloidal anion charge is lowered. For all structures, a reduction in total surface charge shifts the stability window toward longer Debye lengths, consistent with the experimentally observed crystallization trends.

**Figure 5:**
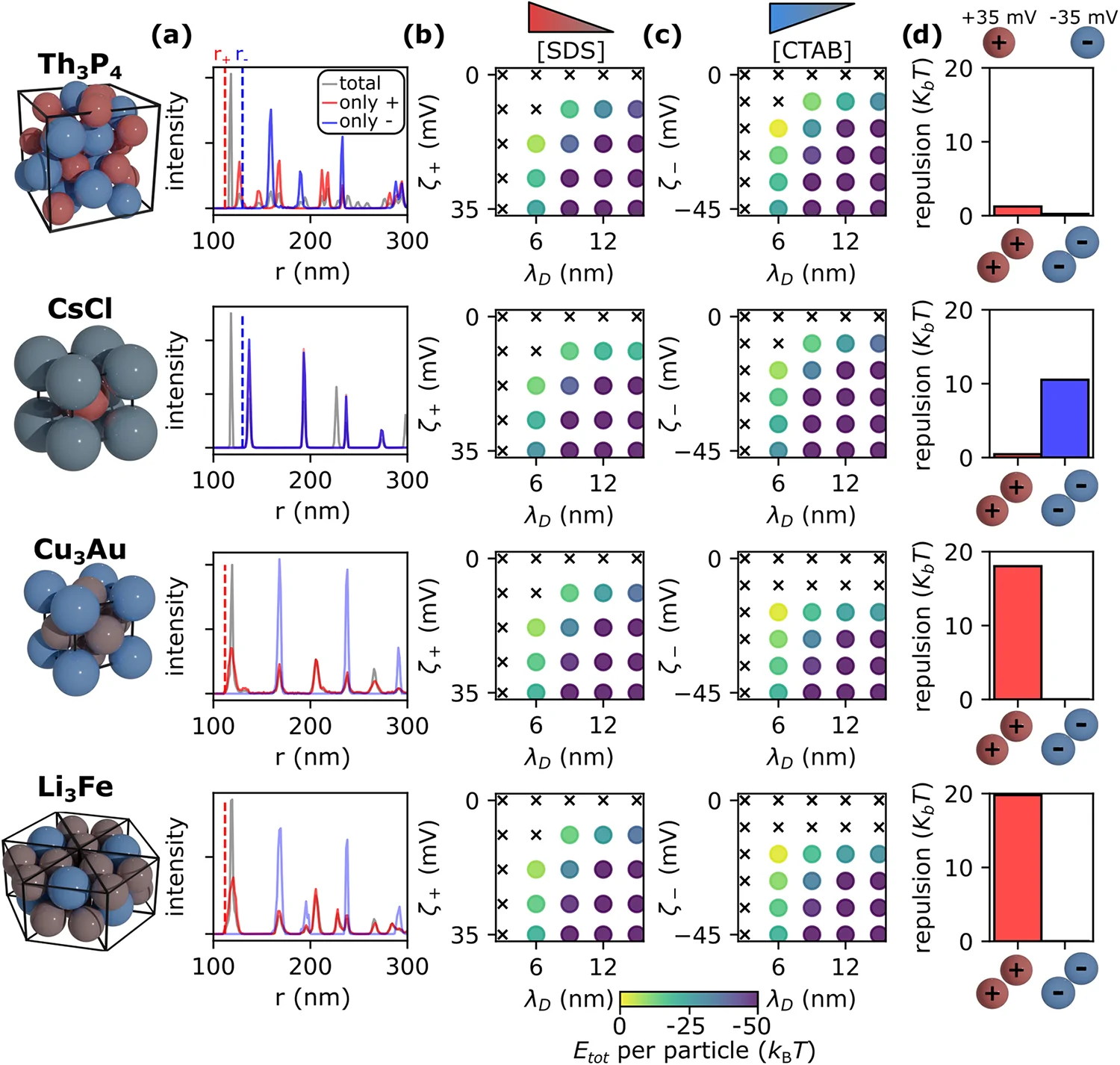
Computed crystal stability based on internal attraction and repulsion. Th_3_P_4_-, CsCl-, Cu_3_Au-, and Li_3_Fe-like crystals (a–d, respectively) are analyzed using a common framework. For each structure (left to right): radial distribution functions after equilibration, with gray showing all particles, red positive particles, and blue negative particles; dotted lines indicate particle radii. Stability phase diagrams as a function of Debye length and surface charge show unstable conditions (crosses) and stable conditions (circles colored by total potential energy per particle). In the first map, the negative particle charge is varied while the positive particles are fixed at +35 mV; in the second, the positive particle charge is varied while the negative particles are fixed at −45 mV. The rightmost panel reports the internal repulsion per particle for positive-positive (red) and negative-negative (blue) interactions at ±35 mV and a Debye length of 12.0 nm. These maps reveal that each lattice is stabilized by reducing the charge of the species that experiences the strongest same-species repulsion.

The simulations further reveal a microscopic origin for these stability regimes (Figure 5a,d). In the CsCl structure, colloidal anions approach each other most closely, leading to significant like-charge repulsion; reducing the anion surface charge therefore stabilizes this lattice. In Li_3_Fe and Cu_3_Au, by contrast, the shortest same-species separations occur between colloidal cations, making their stability highly sensitive to cation–cation repulsion and thus favoring conditions of reduced cation charge. Experimentally, both structures coexist over a range of reduced positive charges; these two structures exhibit similar positive-positive repulsion and energetic stability (Figure 5d). Therefore, their distinct prevalence in experiments likely reflects differences in nucleation kinetics between the two structures.

Finally, Th_3_P_4_ minimizes contact between like-charged species, resulting in weak repulsion and a correspondingly broad stability window. This geometric suppression of like-charge repulsion likely explains the frequent emergence of Th_3_P_4_-like lattices in colloidal and nanoparticle systems.^7,19,27^

Taken together, these experimental and computational results establish a quantitative design rule: for a fixed size ratio, the stable lattice is selected by minimizing the dominant like-charge repulsion, which is controlled by independently tuning the surface charge of each species. Selective and independent tuning of the colloidal cation and anion surface charges, therefore, provides a direct and predictable route to navigating the stability landscape and deterministically selecting among competing crystal structures.

### Crystal Lattice Selection with Other Size Ratios

To examine the generality of this design rule, we extended the experiments to colloidal pairs with different size ratios (β = *r*_small_/*r*_large_). Crystals were grown from pairs with β = 0.53 (400 nm anions and 210 nm cations), 0.60 (240 nm anions and 400 nm cations), and 0.71 (295 nm anions and 210 nm cations), both in the absence and presence of anionic and cationic surfactants. Rather than performing a detailed surfactant titration for each β, we focus here on qualitative lattice selection and use surfactant concentrations that increase the critical crystallization Debye length to approximately 8 nm (≈1.5 mM NaCl). The resulting lattice selections are summarized in the approximate phase diagram in Figure 6a, and detailed characterization of the shown crystals is provided in Supporting Figures S6, S7, and S8.

**Figure 6:**
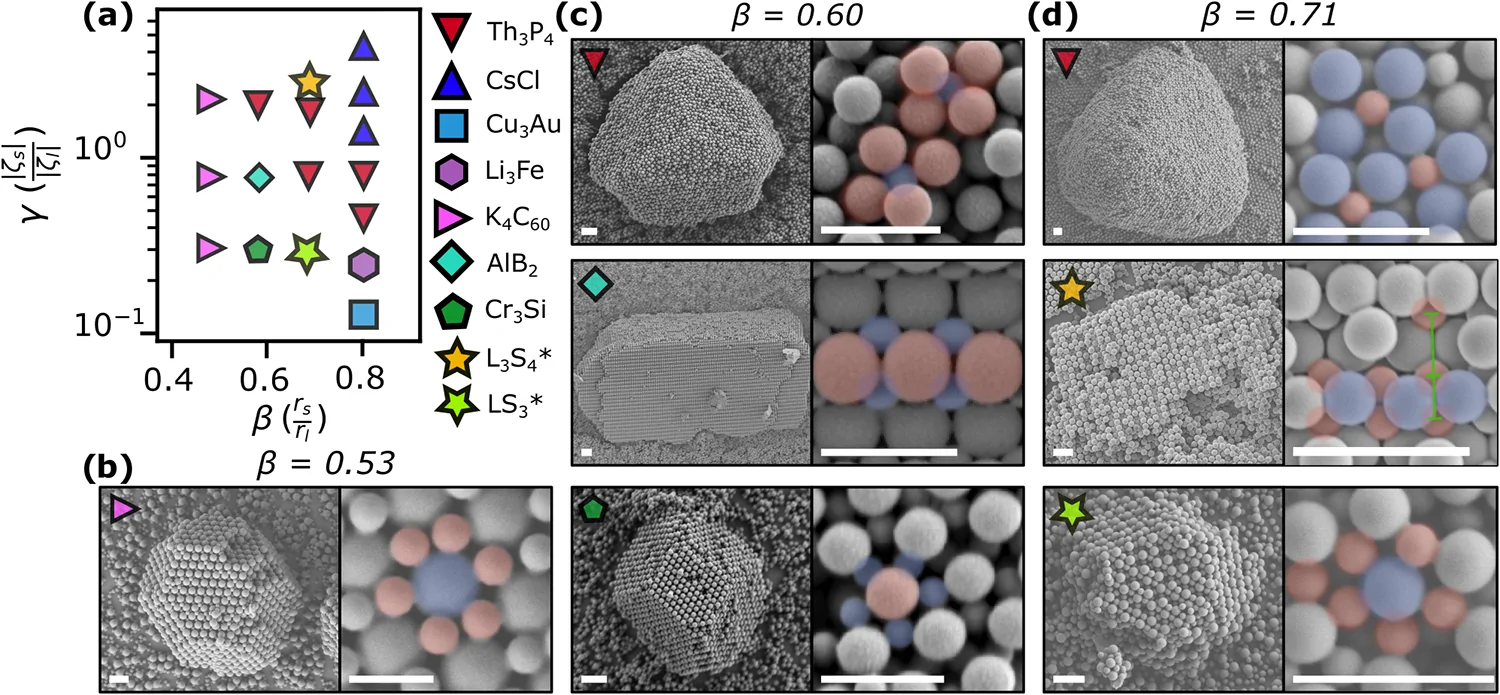
Phase diagram of size ratio (β) and charge ratio (γ) (a) experimental phase diagram showing the observed crystal structures as a function of given β and γ, with γ defined as the ratio between the absolute surface charge of the smaller colloid and the larger colloid. The new crystal structures are shown in SEM micrographs of fixed crystals at low magnification to illustrate the crystal habits (left panels) and at high magnification to show the particle motifs, with positively charged (red) and negatively charged (blue) colloids highlighted in false color (right panels). Two unidentified crystal structures are observed at β = 0.71 and are labeled as L_3_S_4_* and LS_3_*, where asterisks denote tentative assignments. (b) K_4_C_60_-like crystals form at β = 0.53. (c) Th_3_P_4_ (top), AlB_2_ (middle), Cr_3_Si-like (bottom) crystals form at β = 0.60. (d) Th_3_P_4_ (top), L_3_S_4_* (middle), LS_3_* (bottom) crystals form at β = 0.71. All scale bars are 1 μm.

At a β of 0.53, crystals consistent with a K_4_C_60_-like lattice remain dominant under all tested conditions (Figure 6b), including after selective addition of either cationic or anionic surfactant. This does not imply that surfactant-induced lattice selection cannot occur at this size ratio; rather, within the tested stoichiometries and charge conditions, the K_4_C_60_ lattice remains dominant.

More interestingly, we observed significant lattice selection at β of 0.60 and 0.71. For β of 0.60, AlB_2_-like crystals are observed without surfactant (Figure 6c, middle), whereas reducing the charge of the larger cations with SDS results in Th_3_P_4_-like crystals (Figure 6c, top). Conversely, reducing the charge of the smaller anions with CTAB results in Cr_3_Si-like crystals (Figure 6c, bottom), commonly known as the A15 Frank-Kasper phase.^28,29^ Thus, similar to β = 0.85, selectively modifying the charge of either colloidal species provides access to distinct crystal structures from the same pair of building blocks.

While AlB_2_-like crystals have been characterized in previous work,^2^ an in-depth characterization of Cr_3_Si-like ionic colloidal crystals has not yet been reported in the PACS system. In Supporting Figure S7, we provide a detailed characterization of these Cr_3_Si-like crystals, which exhibit a rhombic dodecahedral Wulff structure dominated by {110} facets. Analysis of the small-particle sublattice reveals the characteristic icosahedral coordination environment and three mutually orthogonal particle-chain directions associated with the three orthogonal members of the {100} family, consistent with the Cr_3_Si-like (A15) structure.

For β of 0.71, the relative sizes of the two species are reversed, with the anions now being larger than the cations compared to β = 0.60. In the absence of surfactant, Th_3_P_4_-like crystals are observed (Figure 6d, top). Reducing the charge of the larger anions with CTAB does not suppress the dominant Th_3_P_4_-like lattice, but an additional crystal structure is observed under these conditions. Although its structure cannot be assigned conclusively from the visible planes in the SEM micrographs, the small-particle sublattice exhibits an alternating coupled–separated packing motif. The particle-size ratio is incompatible with an AlB_2_-like structure; we denote this phase L_3_S_4_* and note that it may correspond to the Zangenite lattice reported previously^7^ (Figure 6d, middle). Reducing the charge of the smaller cations with SDS results in no observable Th_3_P_4_-like crystals, while small crystals of a distinct regular dodecahedral habit are observed (Figure 6d, bottom). These crystals exhibit a unit cell that clearly differs from those of both Th_3_P_4_ and L_3_S_4_*, but their structure likewise cannot be assigned conclusively from the available micrographs. Based on the similarity between this surface plane and the characteristic planes of Li_3_Fe- and Cr_3_Si-like structures, we tentatively assign a 1:3 large-to-small particle number ratio to this crystal, which we denote LS_3_*.

The observed lattice selections are consistent with the charge-dependent stability landscape established at a β of 0.85, in which structures are favored where the dominant like-charge repulsion is minimized (the corresponding simulated stability landscape is shown in Supporting Figure S9). In the AlB_2_-like lattice at a β of 0.60, both building blocks experience substantial like-charge repulsion, with the dominant contribution arising from interactions between the smaller anionic colloids. Reducing the charge of the larger building blocks with SDS therefore increases the relative repulsion between the smaller particles, making the AlB_2_-like lattice less stable and favoring Th_3_P_4_-like structures. The Cr_3_Si-like lattice provides a complementary example. Here, the repulsion between both species is relatively weak, but the anion–anion repulsion remains the dominant contribution. Reducing the charge of the smaller species with CTAB consequently reduces the anion–anion repulsion, consistent with the observed formation of Cr_3_Si-like crystals.

At β = 0.71, the two additional crystal structures could not be conclusively identified, preventing a quantitative comparison of their stability and pairwise repulsions. Nevertheless, their observed local packing motifs are qualitatively consistent with the same charge-based picture. Under reduced charge of the larger anions, L_3_S_4_* exhibits closely packed anions, whereas under reduced charge of the smaller cations, LS_3_* exhibits closely packed cations. These observations suggest that selective charge reduction favors structures whose dominant like-charge contacts involve the species whose charge was reduced.

Taken together, these observations show that the lattice selection observed across the different size ratios is consistent with the design rule established at a β of 0.85: the preferred structures minimize the dominant like-charge repulsion. This relationship remains applicable across different particle size ratios.

## Conclusions

We demonstrate that simple ionic surfactants can serve as effective control agents for programming ionic colloidal crystal structures. Selective charge attenuation with CTAB or SDS allows a single pair of oppositely charged colloids to assemble into four distinct lattice types: Th_3_P_4_-, CsCl-, Cu_3_Au-, and Li_3_Fe-like.

Combined experiments and simulations show that this structural selection is controlled by the competition between Coulombic attraction and lattice-specific like-charge repulsion. Th_3_P_4_ is stable across the widest charge window, whereas CsCl-, Cu_3_Au-, and Li_3_Fe-like are selectively stabilized by reducing the charge of the species that experiences the strongest same-species repulsion in each lattice. This establishes charge asymmetry as a predictive and experimentally accessible design parameter for ionic colloidal assembly. Experiments across additional particle-size ratios further demonstrate the generality of this charge-based design rule, with selective surfactant-induced charge attenuation driving the selection between Th_3_P_4_-, AlB_2_-, and Cr_3_Si-like lattices.

These findings provide a general route to programmable colloidal crystals without redesigning the building blocks themselves. More broadly, they highlight how simple molecular additives can unlock structural complexity in colloidal matter and expand access to crystal symmetries beyond those typically realized in atomic ionic solids.

## Methods

### Particle Synthesis and Characterization

Polystyrene (PS) particles were synthesized by surfactant-free emulsion polymerization. Positively charged particles (204 nm diameter) were prepared as follows. A mixture of 550 mL deionized water and 2.5 mL styrene monomer (≥99% MilliporeSigma) was added to a 1 L three-neck round-bottom flask and stirred at 330 rpm using a Heidolph RZR 2020 overhead stirrer. The mixture was purged with nitrogen for 1 h, after which 0.5 g of 2,2′-azobis­(2-methylpropionamidine) dihydrochloride (AIBA, 97% MilliporeSigma) dissolved in 10 mL deionized water was injected. The reaction was then heated to 60 °C and stirred at 330 rpm under nitrogen overnight. A portion of the resulting particles was collected and washed for ζ-potential characterization. The remainder was coated with Pluronic F108 by adding 5 mL of a 5 wt % Pluronic F108 solution, followed by repeated sedimentation-resuspension washing cycles. All particle suspensions were subsequently dialyzed against deionized water for 1 week using 50 kDa Spectra/Por dialysis tubing, with daily water changes to ensure complete removal of residual salt. Negatively charged particles (240 nm diameter) were synthesized identically, except that AIBA was replaced by an equivalent mass of potassium persulfate (KPS, ≥99%, MilliporeSigma) and the styrene volume was increased to 3.0 mL.

For the size ratio study, additional particle pairs were used. The negatively charged 400 nm particles and the positively charged 210 and 400 nm particles were prepared using an identical procedure, with minor adjustments to the styrene volume to control particle size. The negatively charged 295 nm particles were commercially sourced (Microparticles GmbH).

Particle concentrations were determined by gravimetric dry-weight measurements, and particle diameters by scanning electron microscopy (SEM). The ζ-potentials were measured using a Malvern Zetasizer Nano ZS on suspensions containing 0.1 nM colloids, the desired quantity of SDS or CTAB, and 0.5 mM HCl.

### Crystallization Experiments

Two distinct crystallization protocols were employed: static (fixed ionic strength) and dynamic (decreasing ionic strength) conditions.

For static crystallization experiments, suspensions of cationic (3.5 nM) and anionic (2.2 nM) colloids were prepared separately by mixing with Pluronic F108 (0.1 wt %), CTAB or SDS (0–100 μM CTAB or 0–50 μM SDS), and HCl (0.5 mM, to buffer against pH fluctuations). Each suspension was vortexed for 30 s and equilibrated for at least 30 min. The two suspensions were then combined with NaCl solution at the desired concentration (0–3 mM) in a volumetric ratio of 2:1 (cationic/anionic), corresponding to a particle number ratio of 3.2:1, and gently homogenized. The mixtures were loaded into Ibidi-channel slides (sticky-Slide VI 0.4) with a polymer coverslip and sealed. Phase behavior (gas, crystalline, or aggregated) was assessed by bright-field microscopy after 24 h.

For dynamic crystallization experiments, particle suspensions were prepared identically to the static protocol and mixed with NaCl at a concentration at which the particles remain fully stable in suspension (typically 2.2 mM). The suspensions were loaded into flat borosilicate glass capillaries (inner dimensions: 2.0 mm × 0.2 mm, VitroCom, 50 mm length), and one end was sealed with capillary wax. The capillaries were then immersed in a 100 mm diameter Petri dish containing Milli-Q water with the same concentration of SDS or CTAB as in the capillary. Salt ions diffused out gradually through the open end, reducing the ionic strength along the capillary and driving progressive crystallization from the open toward the sealed end. After approximately 5 days, the crystallization front reached the sealed end, at which point the wax seal was removed. The resulting crystals were then fixed by dialysis against surfactant-free Milli-Q water for an additional 7 days to progressively increase the interparticle attraction and lock in the assembled structures. The fixed crystals were then dried on a hot plate at 95 °C, and the capillary was opened by scoring with a diamond-tipped pen.

We also used this dialysis protocol to assemble crystals with different particle-size ratios. For β = 0.71, the HCl and Pluronic F108 concentrations were identical to those in the β = 0.85 protocol. The pre-prepared cationic suspensions were 4.5 nM (final concentration 2.2 nM), and the anionic suspensions were 2.9 nM (final concentration 1.4 nM). The CTAB concentration was 50 μM and the SDS concentration was 25 μM, with an initial NaCl concentration of 1.5 mM. For the samples without the surfactant, the initial NaCl concentration was 4.0 mM.

For β = 0.60, the HCl and Pluronic F108 concentrations were identical to those in the β = 0.85 protocol. The pre-prepared cationic suspensions were 3.2 nM (final concentration of 0.5 nM), and the anionic suspensions were 4.3 nM (final concentration of 0.9 nM). The CTAB concentration was 50 μM, and the SDS concentration was 50 μM, with an initial NaCl concentration of 3.0 mM. For the samples without the surfactant, the initial NaCl concentration was 3.0 mM.

For β = 0.53, the HCl and Pluronic F108 concentrations were identical to those in the β = 0.85 protocol. The pre-prepared cationic suspensions were 4.5 nM (final concentration of 3.4 nM), and the anionic suspensions were 2.9 nM (final concentration of 0.5 nM). The CTAB concentration was 75 μM, and the SDS concentration was 40 μM, with an initial NaCl concentration of 1.5 mM. For the samples without the surfactant, the initial NaCl concentration was 3.5 mM.

### Imaging

Bright-field images were acquired using a Leica DMI3000 inverted microscope equipped with differential interference contrast optics and a high-resolution FLIR Grasshopper3 color camera.

For scanning electron microscopy, opened capillaries were mounted onto aluminum SEM stubs (Ted Pella) using conductive carbon adhesive tabs (Electron Microscopy Sciences) and sputter-coated with 3 nm of platinum. Samples were imaged using a Zeiss MERLIN field emission scanning electron microscope.

### Molecular Dynamics Simulations

MD simulations were performed following previously published protocols using our recently developed PACSim framework,^17,18^ which uses OpenMM^30^ to perform the simulations. For this work, we also contributed a stability workflow to PACSim, which was used for Figure 5 and Supporting Figure S9. The version of the code used for this work and all simulation inputs and data are archived on Zenodo.^31^ All constants in the following equations are given in the provided simulation inputs and are the same as ref 18 unless otherwise specified.

In brief, particles interact using the effective PACS interaction potential given below. In PACSim, each particle has a prescribed radius *R*_*i*_ and a surface charge ψ_*i*_. In this work, ψ_*i*_ is varied to mimic the effect of surfactants.

Electrostatic interactions between the particles (*V*_e_) were modeled using Derjaguin–Landau–Verwey–Overbeek theory,^32^ of the form

$$\frac{V_{e}\left(h_{\mathrm{ij}}\right)}{k_{B}T}=2\pi \epsilon a_{\mathrm{ij}}\psi_{i}\psi_{j}e^{-h_{\mathrm{ij}}/\lambda_{d}}$$

where ϵ is the solvent permittivity (80 ϵ_0_, corresponding to water); *a*_*ij*_ = 2­(*R*_*i*_^–1^ + *R*_*j*_^–1^)^−1^ is the effective radius following the Derjaguin approximation;^32^ λ_d_ is the Debye length; *h*_*ij*_ is the distance between the surfaces of particles *i* and *j*; *k*_B_ is the Boltzmann constant; and *T* is the temperature.

A repulsive potential *V*_p_ prevents particle overlap due to a polymer brush following Alexander-de Gennes theory,^33,34^ and is given by the following equation^35^

$$\frac{V_{p}\left(h\right)}{k_{B}T}=\frac{16\pi a_{\mathrm{ij}}L^{2}\sigma^{3/2}}{35}\left[28\left({\left(\frac{2L}{h_{\mathrm{ij}}}\right)}^{1/4}-1\right)+\frac{20}{11}\left(1-{\left(\frac{h_{\mathrm{ij}}}{2L}\right)}^{11/4}\right)+12\left(\frac{h_{\mathrm{ij}}}{2L}-1\right)\right]$$

where *L* is the polymer brush length and σ is the brush surface density.

For binary particle crystallization simulations shown in Figure 2, systems consisted of 3456 total particles with a positive-to-negative number ratio of 1:1, and a packing fraction of 0.0075. Surface potentials and Debye lengths are specified in the figure and figure captions. Positive particles had a radius of 102.0 nm, and negative particles had a radius of 120.0 nm.

Simulations were performed in a closed system in which particles experienced a shifted Lennard–Jones (SLJ) interaction at distances close to the six walls, following our previous work.^7^ In each direction, the wall interaction potential for a particle of radius *R*_*i*_ within a cutoff distance *r*_*i*_^*c*^ = 2^1/6^ *R*_*i*_ for a shift δ_*i*_ = *R*_*i*_ −1 nm has the form

$$V_{\mathrm{SLJ}}\left(r\right)=4\epsilon \left[{\left(\frac{R_{i}}{r-\delta_{i}}\right)}^{12}-\alpha {\left(\frac{R_{i}}{r-\delta_{i}}\right)}^{6}\right]-4\epsilon \left[{\left(\frac{R_{i}}{r_{i}^{c}}\right)}^{12}-\alpha {\left(\frac{R_{i}}{r_{i}^{c}}\right)}^{6}\right]$$

As the system is extremely dilute, the boundary does not affect the assembly process and only serves to make final crystal identification simpler by avoiding structures wrapped across periodic boundaries.

The potential well depth is set by a parameter ϵ, which is 1 *k*_B_ *T* in the present work, and here we set α = 1, which gives it the form of a Weeks–Chandler–Andersen (WCA) repulsion that goes smoothly to zero at *r*_*i*_ = *r*_*i*_^*c*^ + δ_*i*_.

MD simulations were performed for a maximum of 1 × 10^9^ steps. In Figure 2, the simulations are designated crystalline in the phase diagram by computing the Pearson correlation coefficient of the radial distribution function of the final frame compared to a reference of CsCl or Th_3_P_4_ structure, with a cutoff >0.5 corresponding to a match.

Visualization and analysis were performed using the software Ovito, Blender, and the Python package freud.^36,37^

### Stability Analysis

The *stability* workflow of PACSim attempts to find, for particles of fixed size and charge, a lattice constant for which a particular crystalline arrangement is stable within the PACS potential.

To do so, a preliminary lattice is generated in Python using the coordinates from the primitive unit cell specified through a CIF file. This CIF file can be the one corresponding to an atomic crystalline arrangement obtained from crystal databases.^38^ Particles of specific size and charge are substituted for the atom types in the CIF file as specified through a mapping given in the input file.

The candidate structure is built as an infinite, periodic bulk crystal: the simulation box is the CIF cell scaled to a supercell of specified size, with periodic boundary conditions throughout. The scale is chosen to minimize the total potential energy of the as-built lattice. For a cubic cell a single scale factor suffices, but scaling a lower-symmetry cell uniformly carries the axial ratio of the *atomic* crystal into the colloidal one, which is in general wrong once the particle sizes differ from the atomic radii; for a sufficiently distorted cell this can leave the attractive, opposite-charge pairs far apart while only like-charge pairs are in contact, so that a lattice that should be cohesive reads as net repulsive. We therefore optimize each symmetry-distinct lattice vector independently, grouping vectors by crystal system so that the rescaling cannot lower the symmetry of the cell (a cubic cell keeps one free parameter, hexagonal and tetragonal cells two, orthorhombic cells three).

The lattice is then relaxed at constant temperature under an anisotropic, zero-pressure Monte Carlo barostat, in which each box vector fluctuates independently so that no crystal symmetry is imposed on the final density (20,000 equilibration and 20,000 production steps). The configuration at the mean relaxed box is then energy-minimized; this relaxed, minimized lattice defines the reported interaction energy per particle, *U*_lattice_.

Crystal stability is identified from a Lindemann-type displacement parameter, computed by comparing the last frame of the NPT production trajectory to the as-built (undeformed) lattice–prior to energy minimization, so that thermal displacement, not the minimization step, is what is being measured. For each of the *N* particles, the fractional-coordinate displacement from its as-built position is wrapped by the minimum-image convention and converted to a real-space displacement |Δ**r**_*i*_| using the time-averaged production-tail box. This is averaged over all particles and expressed in units of the nearest-neighbor distance *d*_*nn*_ of the as-built lattice

1 $$L=\frac{1}{N}\sum_{i=1}^{N}\frac{\left|\Delta r_{i}\right|}{d_{\mathrm{nn}}}$$

A lattice is classified as stable for *L* ≤ 0.35.

## Supplementary Material



## Notes

Effect of the F108 brush on CTAB-induced charge neutralization; additional characterization of Cu_3_Au-like crystals; additional characterization of Li_3_Fe-like crystals; photonic band structure of Li_3_Fe and its inverse; effect of number ratio and CTAB on crystal formation; characterization of β = 0.60 colloidal crystals; detailed characterization of Cr_3_Si-like (A15) crystals; characterization of β = 0.71 colloidal crystals; computed stability of β = 0.60 colloidal crystals (PDF)
