# Supplementary material for: Structure Selection in Ionic Colloidal Crystals via Independent Charge Tuning
Source: J Am Chem Soc. 2026 Oct 5;148(41):44091–102. doi: 10.1021/jacs.6c08143 (PMC13693392; doi:10.1021/jacs.6c08143)
Supplement: Supplementary file 1 [file ja6c08143_si_001.pdf]

Electronic Supplementary Information for: Structure Selection in Ionic Colloidal Crystals via Independent Charge Tuning

Steven van Kesteren, Shihao Zang, Cheuk W. Leung, Glen M. Hocky, Stefano Sacanna

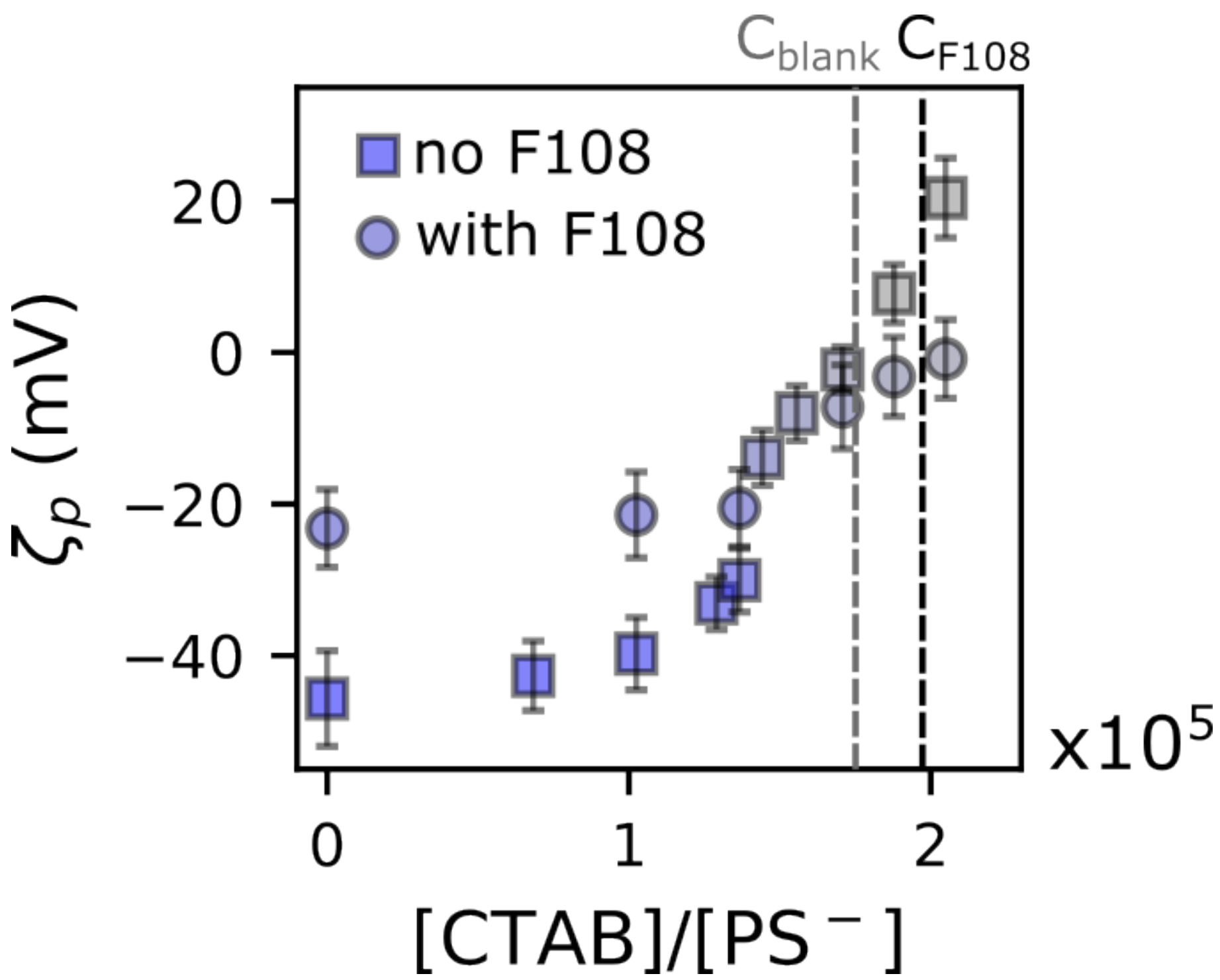

Figure S1: **The effect of polymer brush and CTAB.** The apparent ζ-potential of negative sulfonated 240 nm PS colloids with (circles) and without (squares) a F108 polymer brush as a function of added CTAB. The dotted lines highlight the critical CTAB concentration at which the colloids are neutral. The measured ζ-potential of the F108-coated particles is lower because the surface is partially screened.

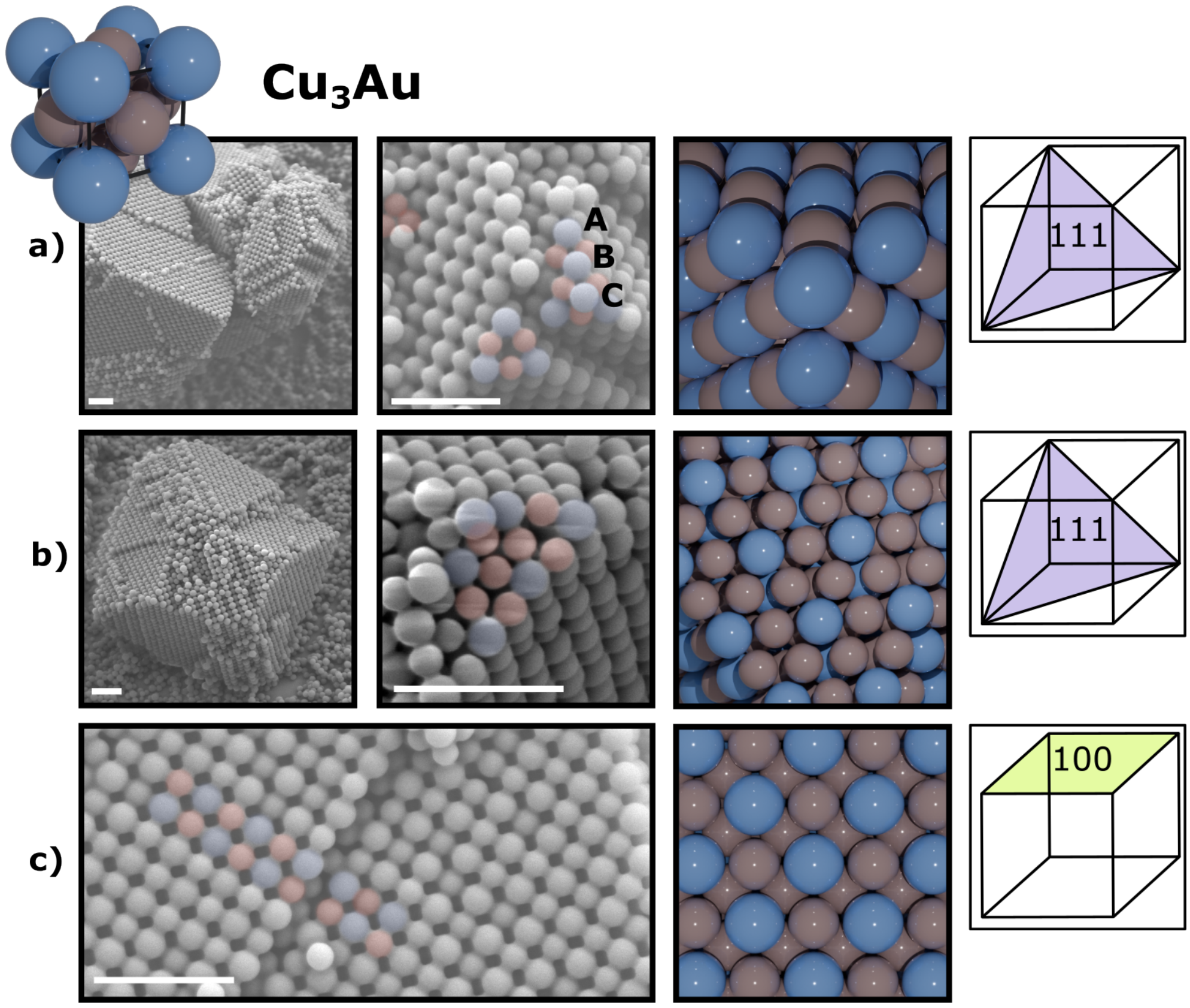

Figure S2: **Additional characterization of Cu_3Au-like crystals. a-c)** SEM micrographs of the positive and negative particles, highlighted by false coloring in red and blue, respectively. Included are 3D renders and a schematic indicating the plane orientation. **a)** Overview (right) and zoom-in (left) of the (111) plane of Cu_3Au-like crystals, highlighting the ABC stacking of these planes. **b)** Overview (right) and zoom-in (left) of the exposed (111) plane of Cu_3Au-like crystals, demonstrating that it is similar to the (0001) plane of Li_3Fe. **c)** The (100) plane of Cu_3Au showing the characteristic checkerboard pattern.

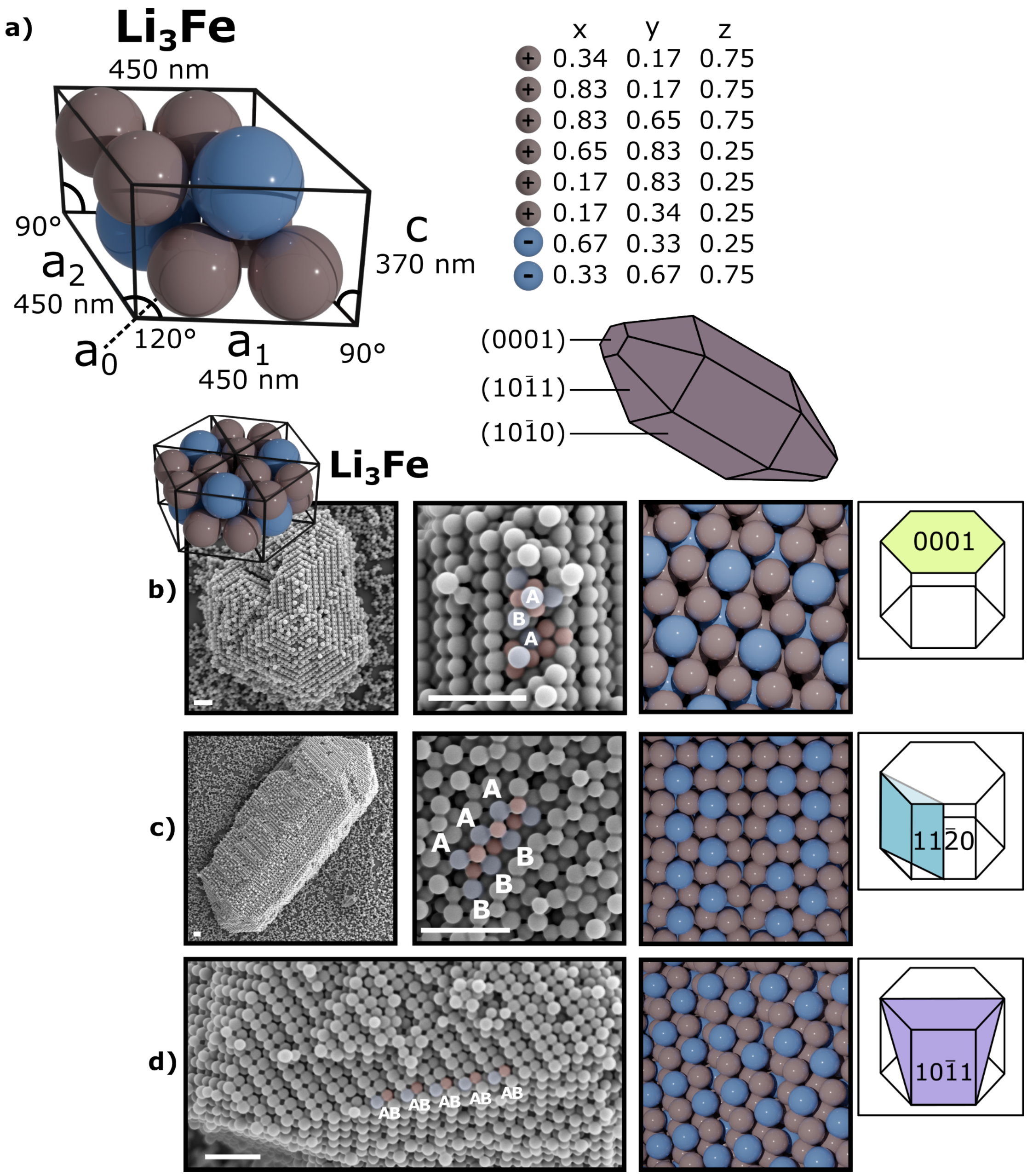

Figure S3: **Additional characterization of Li_3Fe-like crystals. a)** The primitive unit cell of the novel Li_3Fe-like colloidal crystal. Indicating the dimensions (a = 450 nm, b = 450 nm, c = 370 nm) and angles (α = 90°, β = 90°, γ = 120°) of the unit cell (right) and the fractional coordinates of the colloidal ions (left), and a schematic of the prismatic crystal morphology with the Miller indices indicated. **b-d)** SEM micrographs with the positive and negative particles, highlighted by false coloring in red and blue, respectively. Included are 3D renders and a schematic indicating the plane orientation. **b)** Overview (right) and zoom-in (left) of the (0001) plane of Li_3Fe-like, highlighting the ABA stacking of these planes. **c)** Overview (right) and zoom-in (left) of the $(11\bar{2}0)$ plane of Li_3Fe-like, highlighting the ABA zigzag pattern of this plane. **d)** The $(10\bar{1}1)$ plane of Li_3Fe-like, highlighting the AB stair-step stacking of this plane.

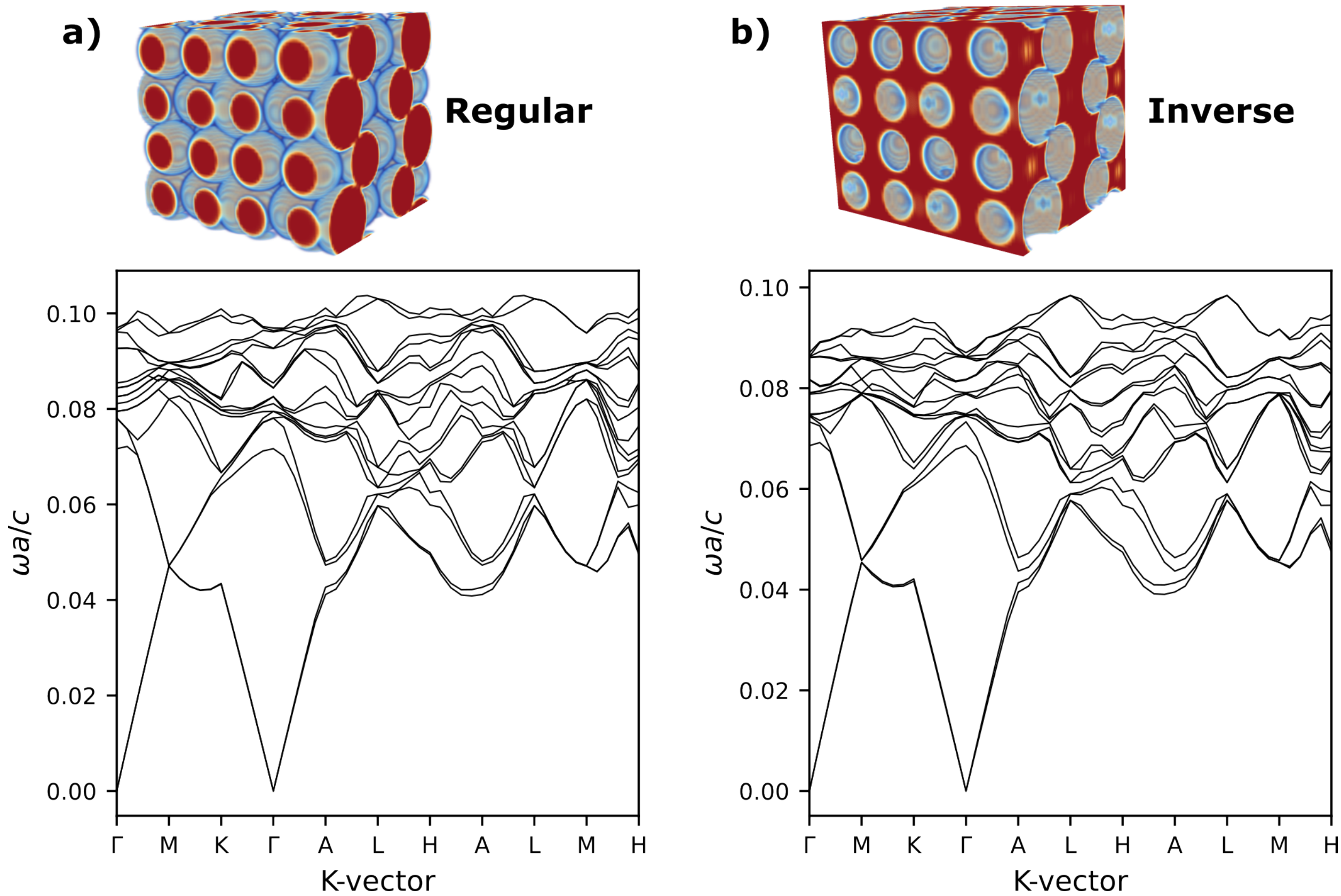

Figure S4: **Photonic band structure of Li_3Fe.** The photonic band structure of Li_3Fe (**a**) and its inverse (**b**) structure for a dielectric constant $\epsilon = 16.0$ for 20 bands calculated using MIT-photonic bands [1]. The K-points are calculated using SeeK-path [2], which finds the K-path.

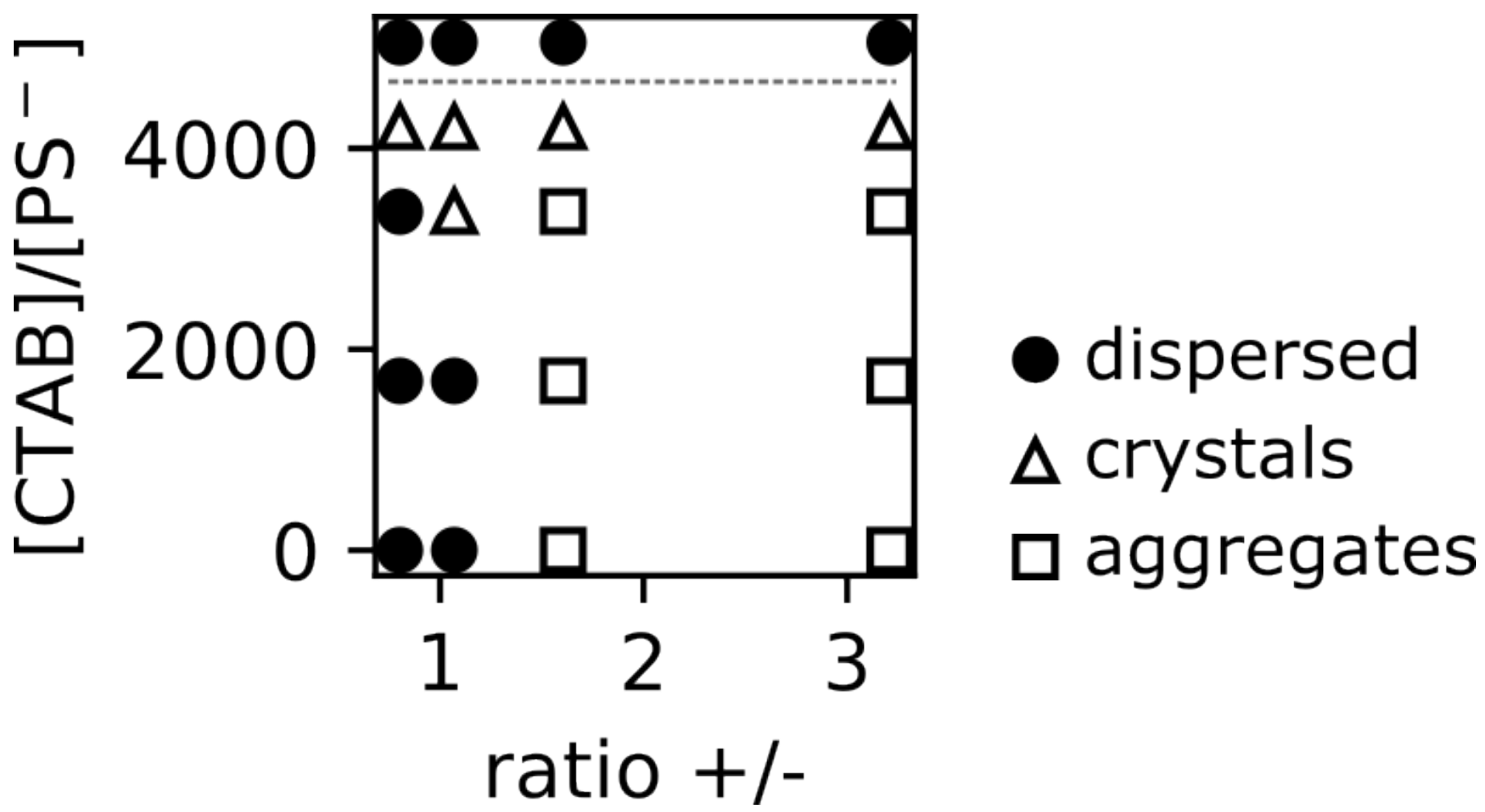

Figure S5: **The effect of number ratio and CTAB on crystal formation.** Experimental phase diagram showing the colloidal gas (circles), colloidal gel (squares), and colloidal crystals (triangles) as a function of positive-to-negative number ratio with different amounts of added surfactant CTAB at a fixed Debye length of 8.0 nm. We observed that, without CTAB, below a ratio of 1.5, no particles assemble; however, with increasing CTAB, crystals begin to form despite the lower total charge.

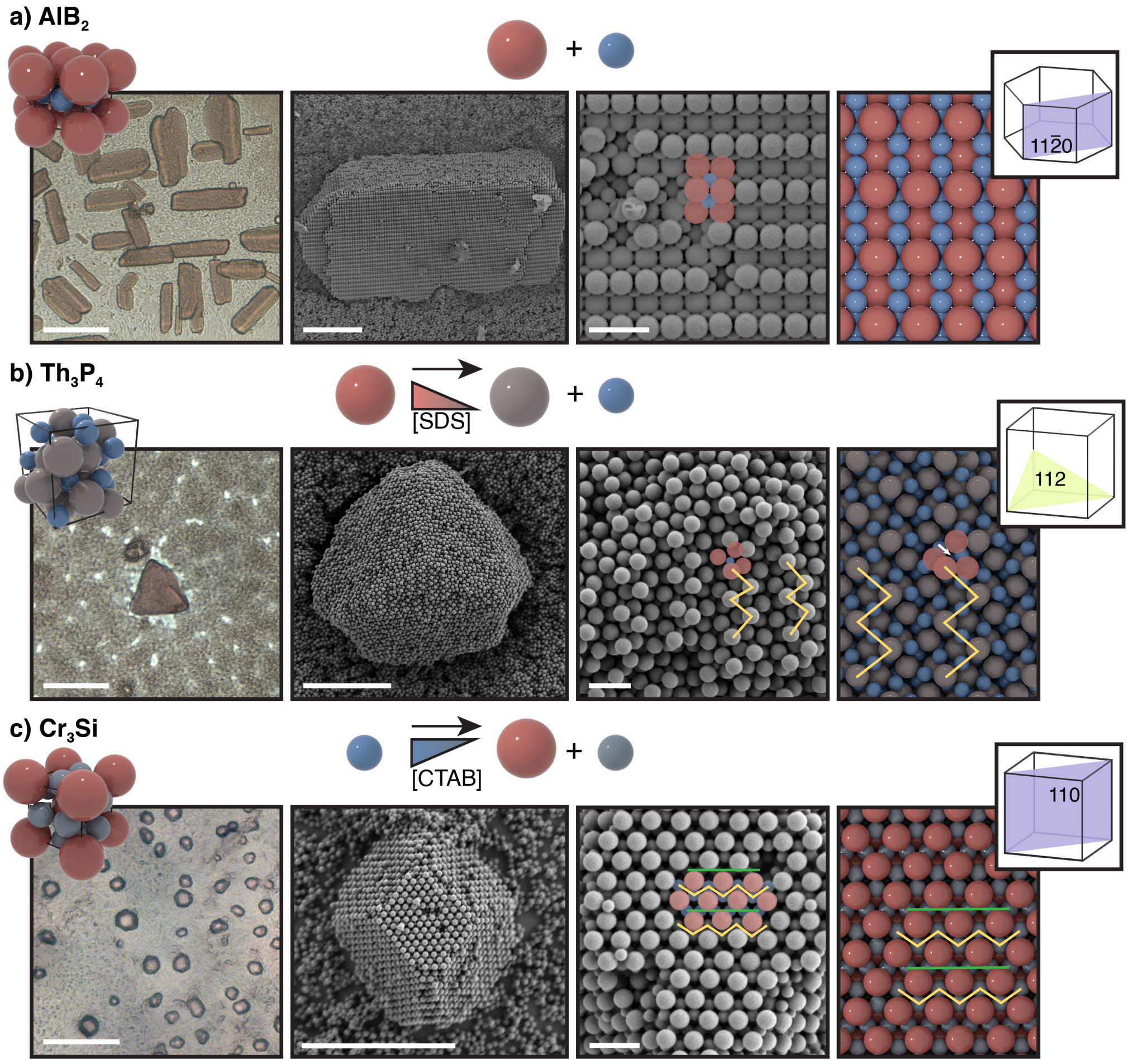

Figure S6: **Characterization of β = 0.60 colloidal crystals.** AlB_2-, Th_3P_4-, and Cr_3Si-like crystals are deterministically assembled from a single mixture of oppositely charged (240 nm (-), 400 nm (+)) colloids by tuning their relative surface charges. For each structure (left to right): unit cell, brightfield micrographs, SEM micrographs of fixed crystals at increasing magnification with positively (red) and negatively (blue) charged colloids highlighted in false color, and 3D renderings of lattice planes corresponding to the SEM images with associated Miller indices. **a)** AlB_2-like lattice formed under no-surfactant conditions is characterized by its $(11\bar{2}0)$ plane. **b)** Th_3P_4-like crystals formed when adding SDS are characterized by their distinct zig-zag arrangement on the (112) plane. **c)** Cr_3Si-like crystals formed when adding CTAB: characterized by the (110)-plane and with more details in Fig. S7. Scale bars: 50 µm (brightfield), 10 µm (low-magnification SEM), and 1 µm (high-magnification SEM).

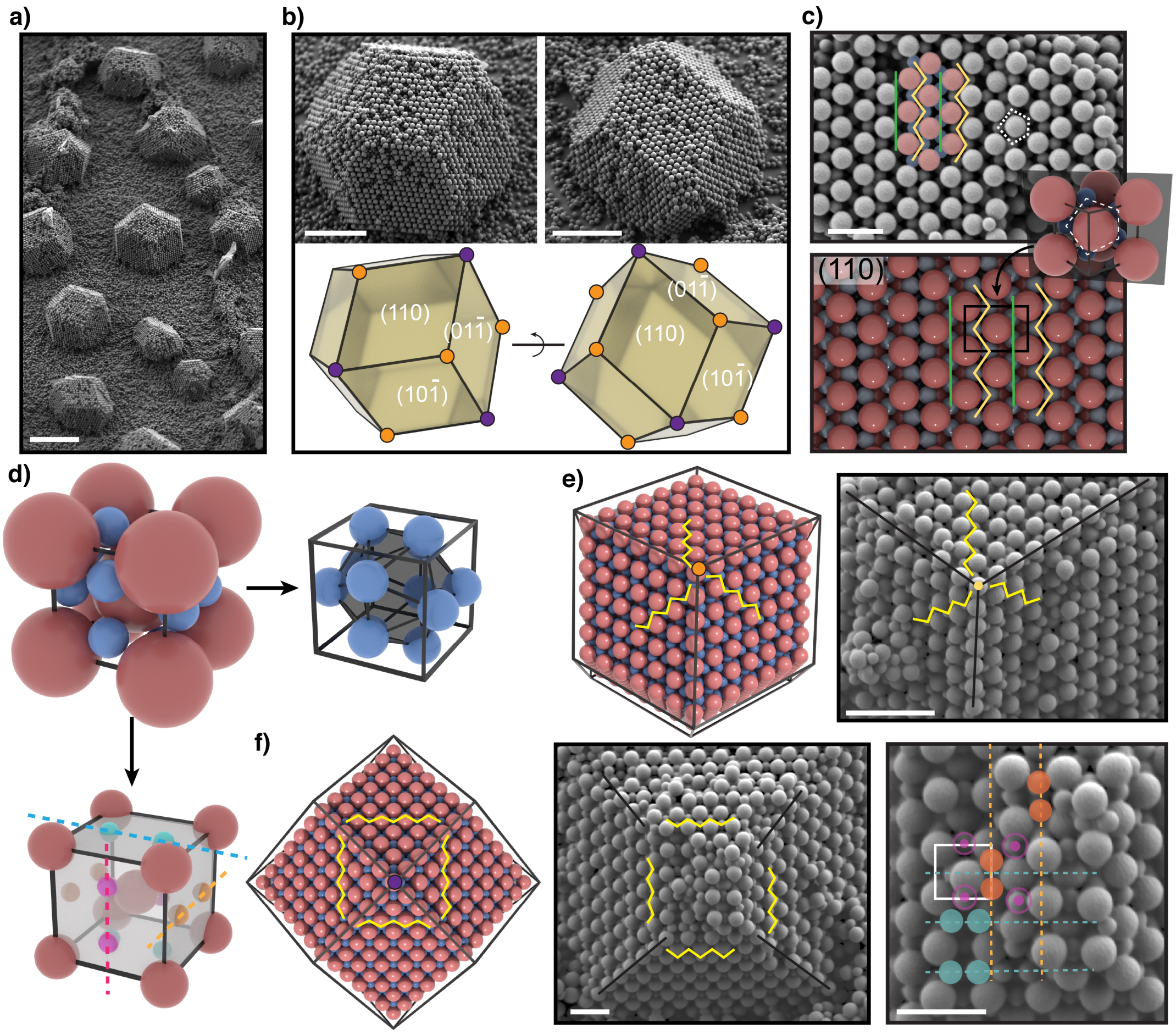

Figure S7: **Characterization of Cr_3Si-like (A15) crystals. a)** Rhombic dodecahedral crystals assembled from (-) 240 nm and (+) 400 nm PS particles in the presence of CTAB. **b)** Rhombic dodecahedra viewed along different orientations, consistent with a Wulff construction dominated by $\{110\}$ facets. **c)** Magnified SEM image of a (110) surface. Small particles in the subsurface layer form characteristic pentagonal motifs (white), which resolve into alternating zigzag (yellow) and linear (green) arrangements. These motifs reproduce the projected packing of the Cr_3Si-like (A15) structure at the (110) plane. **d)** Unit cell of the Cr_3Si-like structure, highlighting the icosahedral coordination environment formed by the small particles (right) and three mutually orthogonal chains of small particles running across the unit-cell faces (bottom). **e)** Orientations of the zigzag motifs on three adjacent $\{110\}$ facets meeting at a $[111]$ vertex (orange), providing a geometric fingerprint of the A15 structure. **f)** Corresponding analysis of the zigzag motifs on four $\{110\}$ facets surrounding a $[100]$ vertex (purple). Their orientations, together with the three mutually orthogonal particle-chain directions on the crystal faces, agree with the unit-cell geometry shown in **d** bottom. Scale bars: **a)**, 10 µm; **b)**, 5 µm; **e)**, 2 µm; **c), f)**, 1 µm.

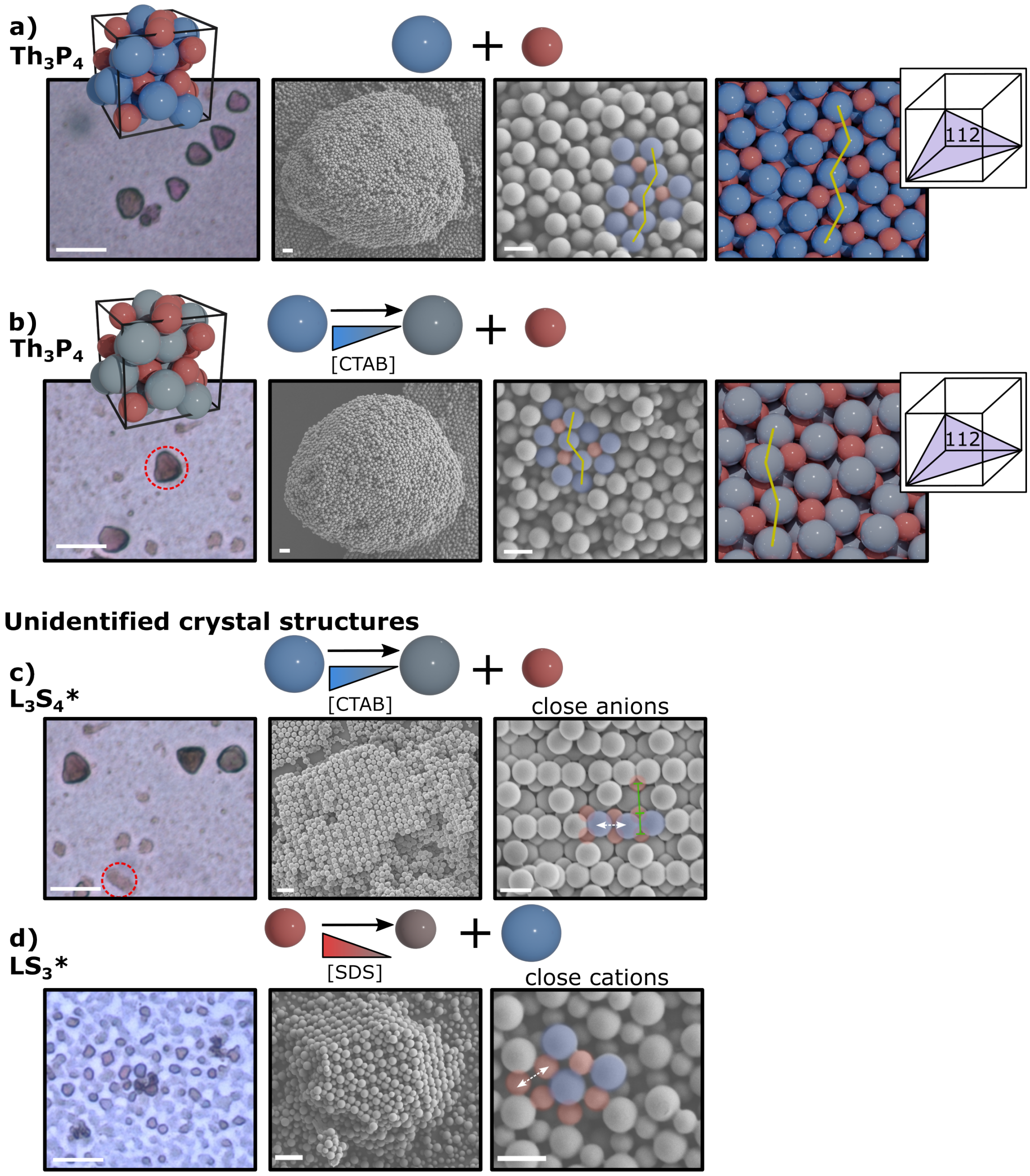

Figure S8: **Characterization of β = 0.71 colloidal crystals.** Th_3P_4-like crystals and two unidentified structures are deterministically assembled from a single mixture of oppositely charged (295 nm (-), 210 nm (+)) colloids by tuning their relative surface charges. For each structure (left to right): unit cell, brightfield micrograph in water, SEM micrographs of fixed crystals at increasing magnification with positively (red) and negatively (blue) charged colloids highlighted in false color, and 3D renderings of lattice planes corresponding to the SEM images with associated Miller indices. **a)** Th_3P_4-like lattice formed under no-surfactant conditions is characterized by its distinct zig-zag arrangement on the (112) plane. **b)** Th_3P_4-like crystals are also formed when adding CTAB. However, a second unidentified crystal is also found under these conditions. **c)** The first unidentified structure ($L_3S_4^*$) shows an alternating coupled–separated packing motif (green) of the small-particle sublattice. **d)** A second unidentified structure (LS_3^*) forms when adding SDS. These crystals share some motifs with Li_3Fe and Cr_3Si lattices. However, neither structure matches the observed habit and crystal planes. Scale bars: 5 µm (brightfield), 1 µm (low-magnification SEM), and 500 nm (high-magnification SEM).

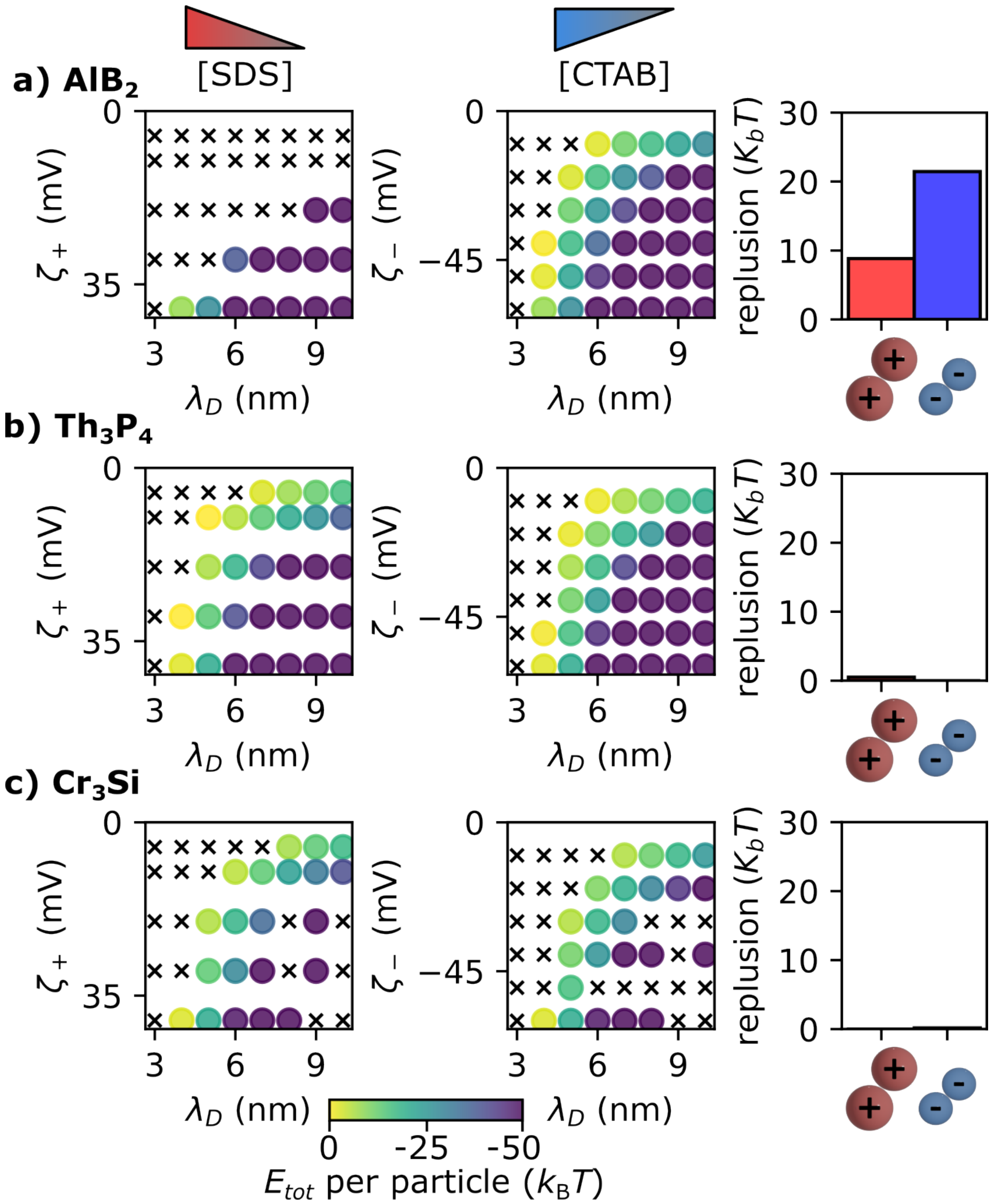

Figure S9: **Computed crystal stability of β = 0.60 colloidal crystals.** AlB_2-, Th_3P_4-, and Cr_3Si-like crystals of $\beta = 0.60$ (**a–c**, respectively) are analyzed using a common framework. For each structure (left to right): Stability phase diagrams as a function of Debye length and surface charge show unstable conditions (crosses) and stable conditions (circles colored by total potential energy per particle). In the first map, the negative particle charge is varied while the positive particles are fixed at +35 mV; in the second, the positive particle charge is varied while the negative particles are fixed at −45 mV. The rightmost panel reports the internal repulsion per particle for positive–positive (red) and negative–negative (blue) interactions at ±35 mV and a Debye length of 12.0 nm. These maps reveal that each lattice is stabilized by reducing the charge of the species that experiences the strongest same-species repulsion. **a)** The AlB_2-like lattice is dominated by anion–anion repulsion. **b-c)** Th_3P_4- and Cr_3Si-like crystals have little repulsion between both species, yet the addition of SDS favors Th_3P_4 (due to having slightly more cation–cation repulsion) and the addition of CTAB favors Cr_3Si-like (due to having slightly more anion–anion repulsion).

References

[1] Steven G. Johnson and J. D. Joannopoulos. Block-iterative frequency-domain methods for Maxwell's equations in a planewave basis. *Optics Express*, 8(3):173–190, January 2001.

[2] Yoyo Hinuma, Giovanni Pizzi, Yu Kumagai, Fumiyasu Oba, and Isao Tanaka. Band structure diagram paths based on crystallography. *Computational Materials Science*, 128:140–184, February 2017.
